# Surface hydrophobic clusters modulate the folding stability and molecular recognition of the disintegrin jarastatin

**DOI:** 10.1016/j.jbc.2025.108294

**Published:** 2025-02-11

**Authors:** Ariana A. Vasconcelos, Russolina B. Zingali, Fabio C.L. Almeida

**Affiliations:** 1 Institute of Medical Biochemistry Leopoldo de Meis (IBqM), Federal University of Rio de Janeiro (UFRJ), Rio de Janeiro, Brazil; 2 National Center for Structural Biology and Bioimaging (CENABIO), Federal University of Rio de Janeiro (UFRJ), Rio de Janeiro, Brazil

**Keywords:** disintegrins, surface hydrophobic clusters, Cleanex, surface forces, oxidative folding, coreless proteins, NMR, water exchange

## Abstract

Disintegrins are cysteine-rich proteins found in snake venoms. These proteins selectively bind to integrins, which play a key role in the regulation of many physiopathological processes. They are coreless proteins that display almost all hydrophobic residues on the protein surface. The exposed hydrophobic residues form surface clusters stabilized by the interaction with the hydrophilic residues in the vicinity and the hydration shell. In the present work, we aimed to determine the stability of surface hydrophobic clusters (SHCs) and their role in protein folding and biological activity. We used urea denaturation curves followed by ^1^H and ^15^N chemical shifts to determine the free energy of unfolding (ΔG_F-U_) and CLEANEX experiments to measure the water exchange rates of the surface amides (k_ex_). The amides with higher local stability and protection from water exchange are those near or at the SHCs, which form a hydrophobic face. SHCs act as foldons, guiding oxidative folding and contributing to the formation of the disulfide bond framework, which is essential for establishing the concave shape and, ultimately, the binding cleft. On the opposite side of the protein are the residues with lower local stability and amides that exchange fast with water. This face coincides with the binding cleft of the protein to the αVβ3 integrin. Taken together, the present work established a correlation between protein hydration and the binding surface.

Disintegrins are a distinct group of proteins known for their high number of disulfide bonds and the unusual exposure of hydrophobic residues on the protein surface. These proteins challenge the classical concept of protein organization, which typically features a well-defined hydrophobic core surrounded by hydrophilic regions that interact with the solvent (1). The absence of a conventional hydrophobic core in disintegrins raises intriguing questions about their stability and solubility, as the exposed hydrophobic residues normally predict a tendency toward aggregation or insolubility in aqueous solutions (2).

In contrast to expectations, disintegrins maintain high solubility in water and generally exist as stable monomers (2, 3). This suggests the presence of compensatory mechanisms that prevent the full exposure of hydrophobic residues to the solvent. Structural analysis revealed that these exposed hydrophobic residues are protected through interactions with spatially adjacent polar residues, concavities on the protein surface, and the shielding effect provided by the solvent (4, 5). These mechanisms give rise to structures known as surface hydrophobic clusters (SHCs), which act as independent foldons capable of conferring stability to the protein even in the absence of a canonical hydrophobic core (5).

The role of the solvent in stabilizing these SHCs is particularly interesting. Solvent shielding not only protects the hydrophobic residues from direct exposure to the aqueous environment but also intensifies the hydrophobic interactions between them through solvation (6, 7, 8). The presence of abundant water around these clusters creates a hydration shell that promotes solvent-induced hydrophobic interactions, similar to the behavior observed in hydrophobic cores, including phenomena such as cold denaturation (5, 6, 9, 10, 11).

Recent studies on plant defensins (4, 5, 12), which share structural characteristics with disintegrins, have revealed that these SHCs exhibit typical properties of canonical mini hydrophobic clusters, suggesting that they act as independent foldons. This discovery opens new possibilities for understanding the role of these clusters in the stability and functionality of disintegrins and other proteins lacking conventional hydrophobic cores.

NMR experiments, such as CLEANEX, enable the mapping of regions of the protein backbone most permeable to water, identifying the backbone amide residues (HN) that exhibit the highest water exchange rates (k_ex_), at timescales of hundreds of milliseconds (13, 14).

From a functional perspective, disintegrins play crucial biological roles through their interaction with integrins (15, 16, 17, 18, 19, 20, 21, 22, 23, 24, 25, 26, 27), which are transmembrane proteins involved in processes such as cell adhesion and signaling. The binding between jarastatin and integrin αVβ3 occurs in a specific binding cleft. A detailed description of this binding interface and the interactions that regulate the affinity and specificity of disintegrins for different integrins is essential for understanding the molecular mechanisms underlying their biological functions (28).

The present work focuses on the characterization and understanding of these SHCs in disintegrins, exploring their contributions to the solubility, stability, and biological function of these unique proteins. The HN of residues that are less prone to exchange with water are those most protected by internal interactions or solvent shielding. This mapping, which was conducted at various pH values, revealed notable segregation between hydrophobic residues and those most permeable to the solvent, which are located on opposite faces of the protein. These findings indicate a clear anticorrelation between solvent-permeable residues and SHCs in disintegrins. These protective and segregated mechanisms are fundamental to understanding the stability of disintegrins, as the spatial arrangement of hydrophobic and hydrophilic residues directly influences their biological function, especially their interaction with integrins.

## Results

**Figure 1:**
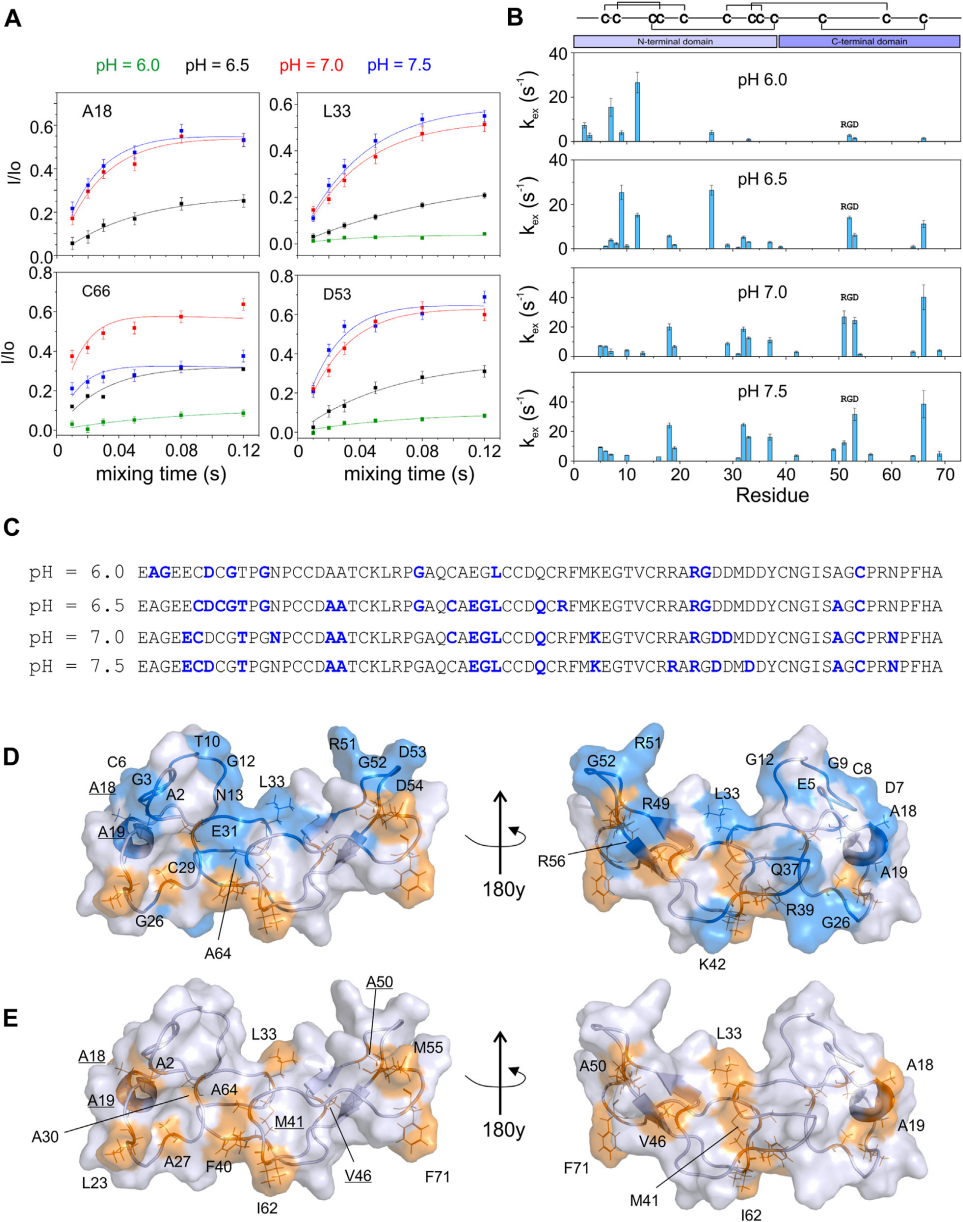
**Measurement of the water exchange rate of jarastatin as a function of pH.***A*, CLEANEX profile for four representative residues (A18, L33, D53, and C66) shown for pH values of 6.0 (*green*), 6.5 (*black*), 7.0 (*red*), and 7.5 (*blue*). All the CLEANEX profiles are presented in Fig. S3. The fitting of the CLEANEX data was performed *via* Equation (4). *B*, water exchange rate (k_ex_) as a function of residue number and pH. *C*, mapping of the residues in fast exchange with water (k_ex_ > 1 s^−1^, in *blue*) for jarastatin at the four pH values. *D*, ribbon and surface representation of the structure of jarastatin showing, in *blue*, a consensus of the residues in fast exchange at the four pH values and, in *orange*, the exposed hydrophobic residues. *E*, ribbon and surface representation of the structure of jarastatin highlighting the exposed hydrophobic residues.

Our goal was to describe the solvent interactions at the surface of jarastatin and correlate them with the distribution of the hydrophobic residues exposed to water. For that, we used the CLEANEX experiment as a way to pinpoint the regions at the protein backbone most susceptible to water exchange. CLEANEX enables the measurement of the water exchange rates (k_ex_) of the backbone amide hydrogen (HN) on the timescale of milliseconds (13, 14). The HN of residues that are more protected from water exchange (k_ex_ < 1 s^−1^) are not detected by CLEANEX. To better access the residues in fast exchange with water, we measured CLEANEX at four different pH values, ranging from 6.0 to 7.5 (Fig. 1*A*), since at higher pH values, an increase in k_ex_ is expected. Notably, at pH 6.0, the residues in fast exchange with water are located mainly in the N-terminal domain of jarastatin (residues 1–38). At higher pH values, we could better access residues in the C-terminal domain (residues 39–73), including the RGD loop (Fig. 1*B*). The greater accessibility of water to the backbone of the N-terminal domain is expected because of its high plasticity. Previous reports have shown that the N-terminal domain displays many residues involved in conformational exchange at the microsecond timescale and motion from pico-to nanosecond, mainly in residues at the N-terminus (1–10), C-terminus (68–73), and RGD loop (51–56) (28, 29).

Interestingly, we observed that the residues in fast exchange with water are segregated to a protein face (Fig. 1*D*) that is opposite to the face containing the majority of the hydrophobic residues, which we refer to as the hydrophobic face (Fig. 1*E*). Remarkably, this face with the HN in fast exchange with water coincides with the binding cleft mapped for interaction with the integrin αVβ3 (28). One possible interpretation of this observation is that the hydrophobic side chains are protected from direct exposure to water by the SHCs, which are dynamic surface elements where the solvent-exposed hydrophobic residue is protected by the adjacent polar side chains and the shielding of protein solvation (Fig. S2) (4, 5, 12). The hydrophobic face contains most of the residues, with their HN protected from exchange with water; consequently, they were not detected by CLEANEX. Fig. S3 shows all the CLEANEX data at several pH values.

**Figure 2:**
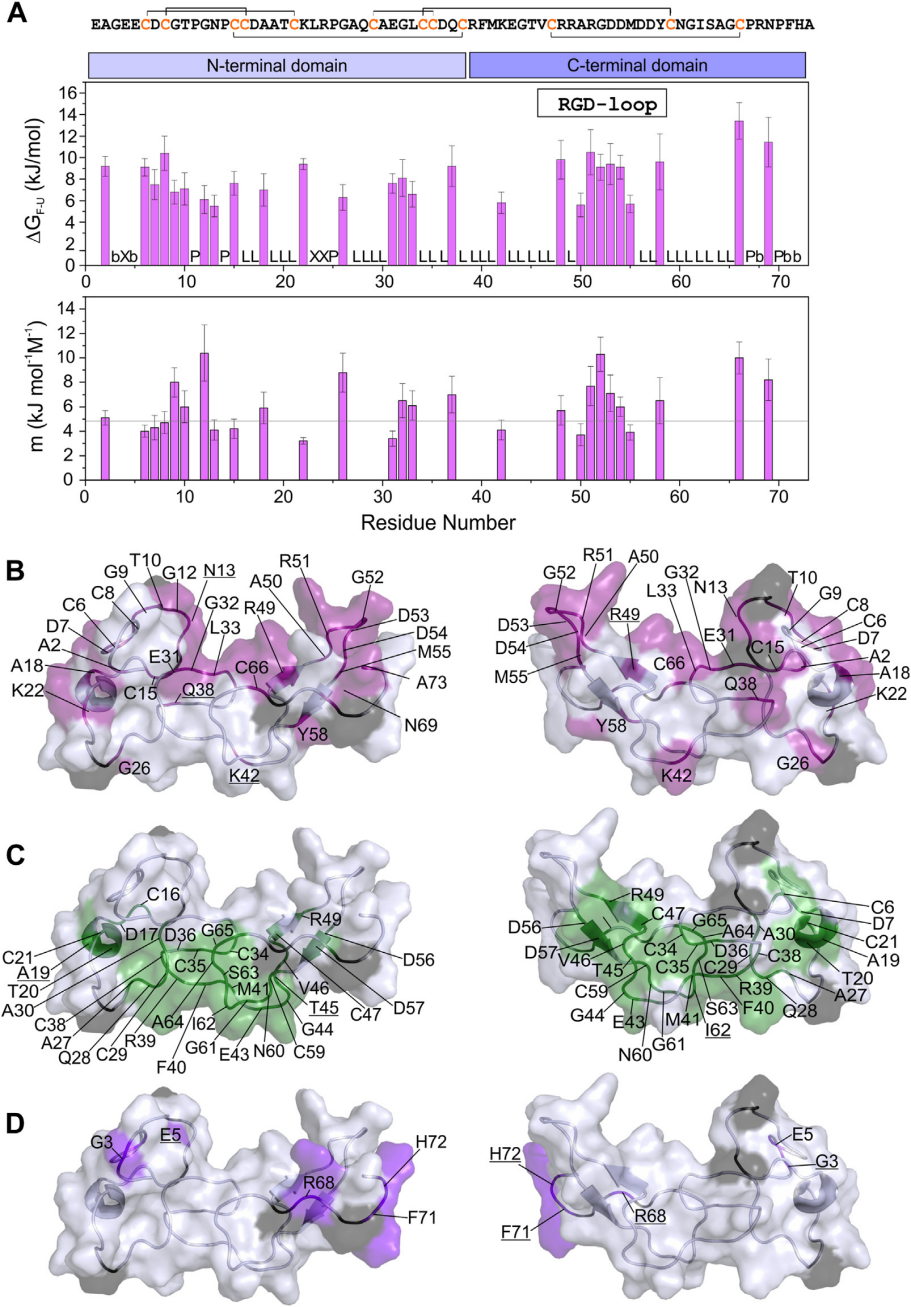
**The local free energy of unfolding (ΔG**_**FU**_**) and m value were obtained from the urea denaturation profiles (**Fig. S4**).***A*, ΔG_FU_ and m as a function of the residue number. These thermodynamic parameters were calculated only for the residues that presented a sigmoidal profile. The residues with a linear profile up to 4 M urea (L) were interpreted as the most stable on the protein surface since full unfolding was observed at concentrations higher than 4 M urea. The residues labeled “P” are prolines, as “X” represents the residues with no information because of the lack of assignment, and “b” represents the residues that vanished in the presence of urea. *B*, ribbon and surface representation of the structure of jarastatin, highlighting the residues with low or intermediate stability that showed a sigmoidal profile upon urea denaturation. *C*, ribbon and surface representation of the structure of jarastatin, highlighting the residues with higher stability that showed a linear profile upon urea denaturation. *D*, ribbon and surface representation of the structure of jarastatin highlighting the residues that vanished upon urea denaturation.

The observations described above provided the opportunity to correlate the water exchange of each face of the protein with the local structural stability. With this goal, we measured the stability of jarastatin *via* urea denaturation, following the chemical shifts of the backbone ^1^HN and ^15^N by NMR at several urea concentrations (Figs. 2, S4). We observed a sigmoidal curve of the chemical shift perturbation as a function of the urea concentration for 26 residues (up to 4 M urea) (Fig. S4). From these data, we obtained the free energy of unfolding (ΔG_F-U_, Fig. 2*A*) and the *m* value (Fig. 2*B*). The other 32 residues displayed a linear profile up to 4 M urea. These residues were interpreted as the most stable residues on the protein surface since full unfolding would be observed at concentrations higher than 4 M urea (Fig. 2*A*, labeled L). Few residues vanished in the spectra upon increasing the urea concentration, probably because of extreme broadening due to conformational exchange (Fig. 2*A*, labeled b).

Each of these residues was mapped to the structure of jarastatin. We observed that the same face that showed fast water exchange (Fig. 1*D*, blue) presented a sigmoidal denaturation profile, indicating low to intermediate local stability, with ΔG_F-U_ values between 5 and 16 kJ/mol (Fig. 2, *A* and *B*). The residues at the opposite face showed a linear profile and were considered to present higher local stability, with ΔG_F-U_ higher than ∼20 kJ/mol. This face coincides with the face containing most of the hydrophobic residues, as shown in Figure 1*E*. In summary, HNs that are more susceptible to urea denaturation are in surface regions that are more permeable to water, whereas more stable regions are at the hydrophobic face. Notably, most of the residues with low and intermediate local stability are in the N-terminal domain and in the RGD loop at the C-terminal domain of jarastatin (Fig. 2*A*).

We also obtained the *m* value from the fitting of the sigmoidal denaturation curves (Fig. 2*A*). The *m* value varied from 3 to 12 kJ mol^−1^M^−1^. The *m* value correlates with the amount of protein surface exposed to solvent upon unfolding (30). We observed an average of 4.8 kJ mol^−1^M^−1^ for the *m* values obtained for jarastatin, which is compatible with the exposed surface upon unfolding expected for a protein with 73 residues (30).

**Figure 3:**
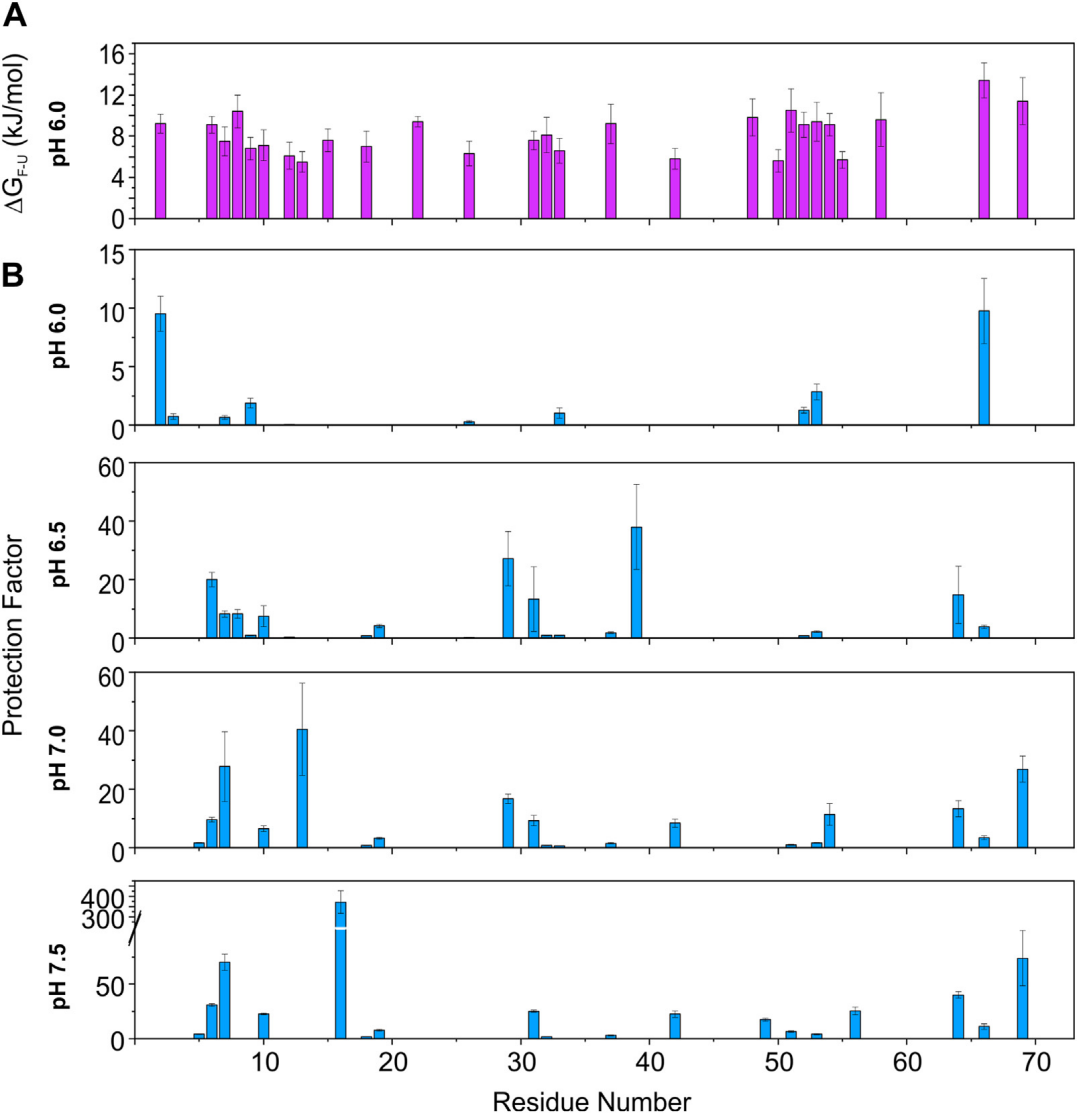
**Comparison of the local stability of the fast exchange amides with that of the water exchange protection factor jarastatin.***A*, for comparison, we plotted the ΔG_FU_ as a function of the residue number, as shown in Figure 2. Note that in this plot, we considered the amides that were undetectable by CLEANEX with ΔG_FU_ > 20 kJ/mol. *B*, protection factor for each amide proton measured from CLEANEX experiments. Note that in this plot, we considered the amides that were undetectable by CLEANEX with a protection factor greater than 480.

We calculated the protection factor for each HN at various pH values and compared it with that of ΔG_F-U_ (Fig. 3). We observed a correlation, showing that the regions of least protection are those with lower ΔG_F-U_ at pH 6.0. The correlation is very clear at pH values of 6.0 and 6.5, for which the residues with low protection factors coincide with the residues with low ΔG_F-U_ values. Other contributions must be considered at pH values of 7.0 and 7.5, such as changes in ΔG_F-U_ due to changes in pH. As the pH increases, alkaline conditions can promote local protein unfolding, which exposes the amide groups to the solvent. This exposure makes the amide groups more susceptible to proton exchange with water, thereby reducing the protection factor (*i.e.*, the resistance to hydrogen exchange). Generally, the more exposed these groups are to the solvent and the less structured these groups are, the lower the protection factor will be.

**Figure 4:**
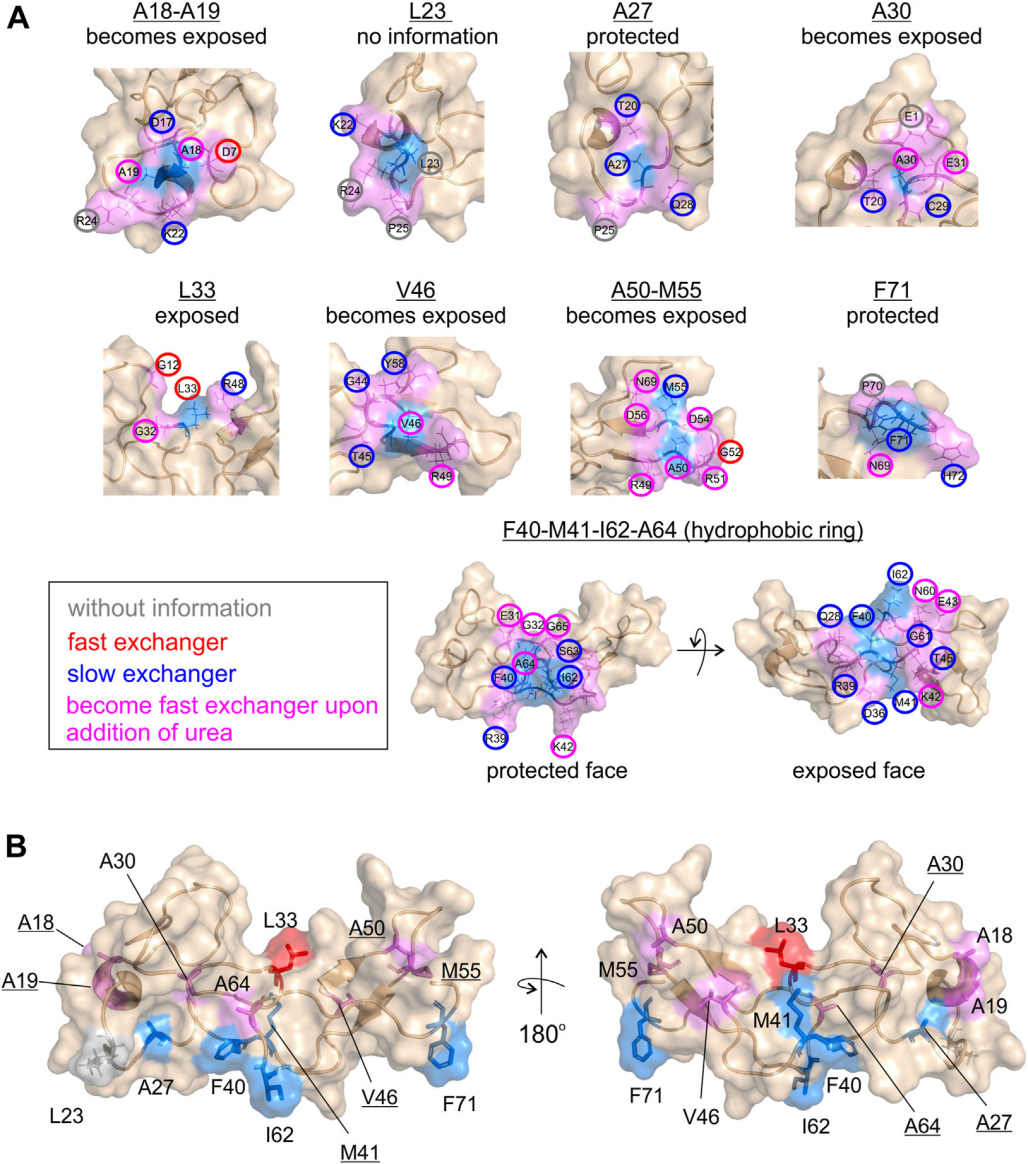
**Water exchange properties of the surface hydrophobic clusters of jarastatin.***A*, surface hydrophobic clusters highlighting the exposed hydrophobic residues in *blue* and the spatially adjacent residues in *violet*. The circles show the residues classified as “fast exchangers” (*red*), when they were detectable by CLEANEX in the absence of urea, “slow exchangers” (*blue*), when they were not detectable by CLEANEX even in the presence of urea, “becomes fast exchangers upon the addition of urea (*magenta*), and when there is not enough information (*gray*). On the basis of their water exchange behavior, each cluster was classified as exposed (less stable), exposed upon the addition of urea (intermediate stability), or protected. *B*, ribbon and surface representation of jarastatin highlighting the exposed hydrophobic clusters according to their behavior. The *blue dots* are the most stable (protected), the *red dots* are the exposed (less stable), and the *magenta dots* are the ones with intermediate stability (exposed upon the addition of urea).

We now want to understand how the hydrophobic face, which is the region with the highest local stability, behaves upon urea denaturation. To obtain this information, we measured the water exchange rates of jarastatin as a function of the urea concentration up to 4 M (Tables S1 and S2, Fig. 4). We observed four different behaviors.

1- Amides exposed to water exchange even without the addition of urea are fast water exchangers, which are residues that are observable in CLEANEX in the absence of urea. They maintain fast exchange upon the addition of urea. Some of these fast exchangers became undetectable in the CLEANEX at higher concentrations of urea, possibly because the full exposure to the solvent made the exchange too fast to be detected; therefore, the signal may have become too weak due to rapid saturation transfer. Another possible reason is the occurrence of conformational exchange induced by urea, which induces line broadening.
2- Amides that become exposed upon the addition of urea—these residues are undetectable by CLEANEX in the absence of urea due to slow water exchange and turn into fast exchangers upon the addition of urea. The amide protons of these residues became solvent accessible at higher urea concentrations, demonstrating local destabilization.
3- Amides protected from water exchange (k_ex_ < 1 s^−1^) are slow exchangers, which are undetectable by CLEANEX at all measured urea concentrations. These residues are the most urea-resistant residues and present amide protons protected from water exchange up to 4 M urea.
4- Amides that are exposed to water exchange but undetectable by CLEANEX owing to fast exchange rates in which the signal may become too weak owing to rapid saturation transfer. This behavior was observed especially at pH 7.5. We interpreted amide protons that were exposed to water (fast exchanger) or became exposed to water upon the addition of urea at pH 6.0 and were undetectable by CLEANEX at pH 7.5 as undetectable fast exchangers (Tables S1 and S2).

We summarized the effect of urea on k_ex_ at pH 6.0 (Table S1) in the SHCs in Figure 4*A*. Each hydrophobic residue and the spatially adjacent polar residues were highlighted according to their behavior. This analysis enabled the classification of each of the SHCs as follows: 1) protected amide protons even in the presence of urea, which are the most stable clusters; 2) exposed upon the addition of urea, which are the clusters of intermediate stability; 3) exposed in the absence of urea, which are the least stable clusters; and 4) with insufficient information. We then identified the classified SHCs in the jarastatin structure (Fig. 4*B*). This analysis finely tuned the segregation observed earlier, where the highly stable SHCs are in one face (A27, F40, M41, I62, and F71), and the less stable clusters in a lateral (A18, A19, A30, A50, M55, A64) or opposing face (L33), which coincides with the jarastatin binding site to αVβ3 (28). Notably, L33 is the least stable surface hydrophobic cluster, and it is at the center of the binding cleft.

These data are in accordance with the previous analysis, confirming the segregation of the protein in one face containing the fast exchangers (binding cleft) and the opposite face containing the slow exchangers, which contain most of the hydrophobic residues (Fig. 1). The hydrophobic face contains residues with the highest stability upon urea denaturation (highest ΔG_F-U_, Fig. 2) and more stable SHCs (Fig. 4*B*).

Next, we evaluated whether urea exclusively affects local protein stability or whether there is differential access of urea to specific protein regions. To address this question, we performed ^15^N-edited NOESY-HSQC and ROESY-HSQC at a short mixing time (20 ms).

One way to study solvation is to directly measure, *via* NMR, the dipolar coupling between solvent molecules (water or urea) and protein hydrogens. This method was first proposed by Otting *et al.* (1991) (31, 32), where NOEs measured in the laboratory frame (NOESY) or the rotating frame (ROESY) provide information about the typical residence times of water or urea. Short mixing times are used to minimize contributions from auto relaxation and spin diffusion. The measured NOE intensities directly reflect cross-relaxation rates in either the laboratory or rotating frame.

A negative NOE/ROE intensity ratio (negative NOE and positive ROE) indicates the presence of tightly bound water interacting with protein HNs. Tightly bound solvent molecules (water or urea) exhibit rotational correlation times on the order of nanoseconds. In contrast, highly dynamic solvent molecules, which interact transiently with the protein, have rotational correlation times on the order of picoseconds.

**Figure 5:**
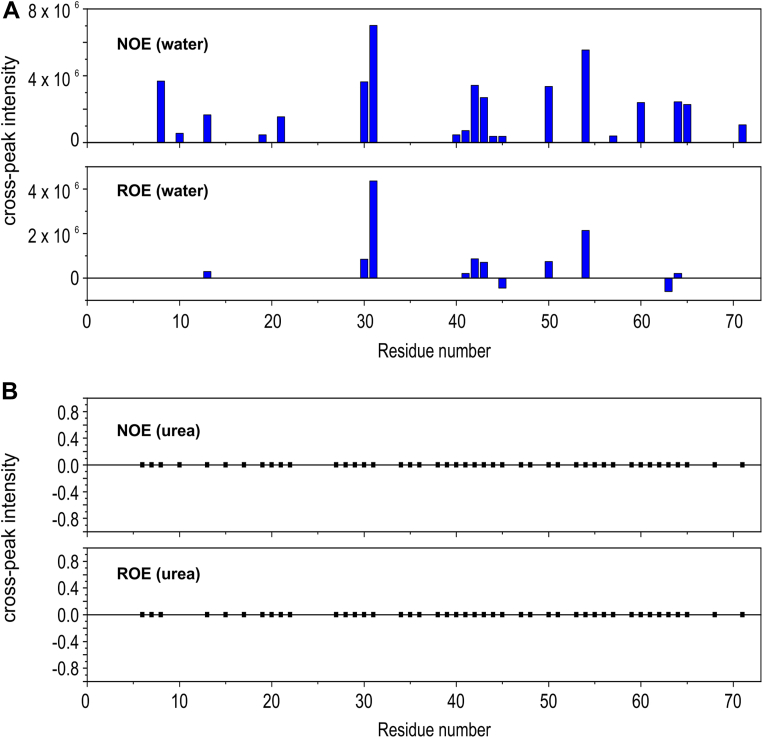
**Hydrogen nuclear spin dipolar interaction of water and urea with jarastatin.***A*, intensities of the NOE and rotating-frame NOE (ROE) between water and each amide hydrogen of Jarastatin at a short mixing time (20 ms). *B*, intensities of the NOE and ROE between urea and each amide hydrogen of jarastatin at a short mixing time (20 ms). Note that no NOE or ROE was observed with urea at this mixing time.

In the case of jarastatin (Fig. 5), we observed numerous NOEs with water but none with urea. This demonstrates that interactions with water are predominant, whereas interactions with urea are much less significant. This finding suggests that urea most likely does not preferentially access any specific region of the protein. Furthermore, since the observed ROEs and NOEs have the same phase (positive), the solvation shell is highly dynamic, with water residence times on the order of hundreds of picoseconds. The negative ROEs (T45 and S43), which are antiphase relative to their diagonal cross peaks, reveal chemical exchange rather than dipolar interactions.

We conclude that the differential sensitivity of specific residues to urea is due primarily to changes in local stability at the protein surface rather than preferential access of urea to specific regions.

The protein region with the highest stability is the one that contains most of the SHCs, forming a scaffold that, along with the disulfide bonds, maintains the concave shape that composes the binding cleft (28). We used the fluorescent probe 4,4′-dianilino-1,1′-binaphthyl-5,5′-disulfonate (bis-ANS) as a tool to measure the presence of exposed hydrophobic patches and their stability to chemical denaturation by urea. The fluorophore bis-ANS is a molecular probe widely used to study the exposure of hydrophobic regions in proteins (33, 34). Its mechanism of action is based on its ability to bind to hydrophobic surfaces exposed during processes such as protein denaturation or oligomerization. In aqueous solution, bis-ANS exhibits low fluorescence; however, when it is associated with ordered exposed hydrophobic regions of proteins, its fluorescence increases significantly.

In the chemical denaturation profile of a globular protein, low bis-ANS fluorescence is expected in the absence of a chemical denaturant. As the concentration of the denaturant increases, the fluorescence increases due to the exposure of the ordered hydrophobic surfaces. However, with denaturant concentration increasing even further, the fluorescence decreases because bis-ANS is unable to bind to disordered exposed hydrophobic surfaces (33, 34).

**Figure 6:**
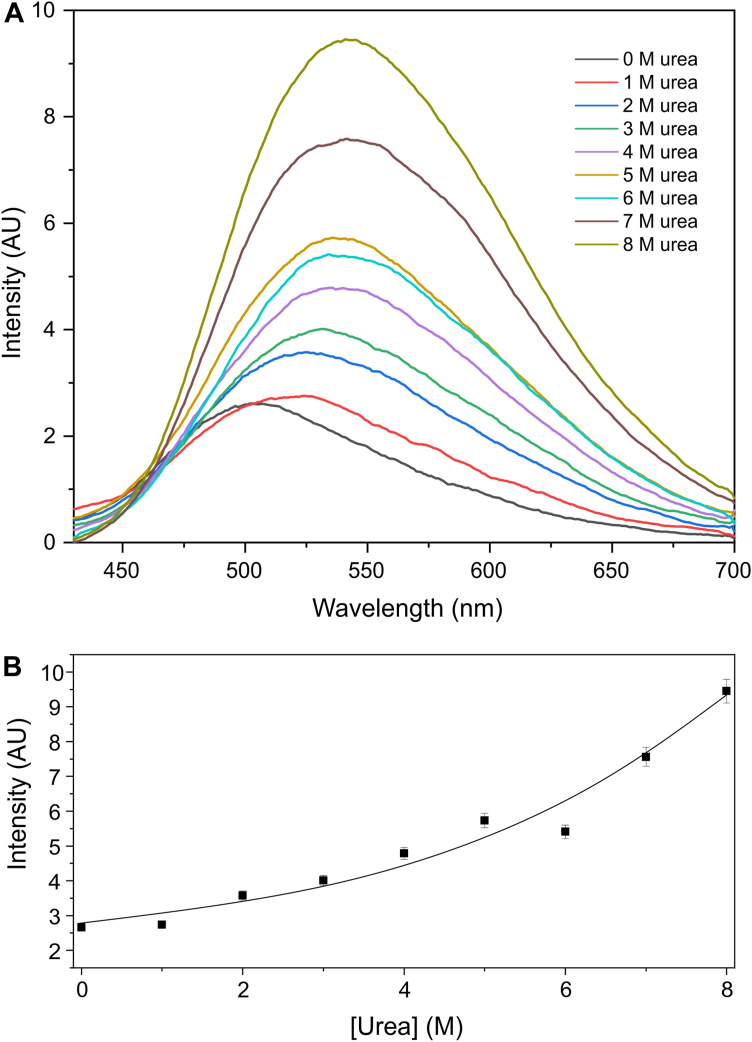
**Exposed hydrophobic surface measured by the fluorophore bis-ANS.***A*, differential fluorescence emission spectra of 5 μM bin-ANS in the presence and absence of 5 μM jarastatin as a function of the urea concentration. *B*, maximum fluorescence emission intensity as a function of the urea concentration.

We measured the effect of urea-induced denaturation up to 8 M by monitoring bis-ANS fluorescence emission (Fig. 6). The figure presents the difference spectra of bis-ANS fluorescence in the presence and absence of an equimolar concentration of jarastatin (5 mM). A significant increase in bis-ANS fluorescence emission was observed, even in the absence of urea (Fig. 6*A*, black), confirming the presence of ordered exposed hydrophobic surfaces in jarastatin. The addition of urea, up to 8 M, resulted in an exponential increase in fluorescence emission (Fig. 6*B*), indicating greater exposure of the ordered hydrophobic surfaces. Interestingly, a decrease in fluorescence emission, typically associated with fully denatured states (disordered hydrophobic exposure), was never observed. This suggests that the hydrophobic surface clusters are highly stable and that even 8 M urea is insufficient to fully denature them. This finding aligns with the linear chemical shift perturbation profile observed for the hydrophobic face under urea denaturation (Fig. 2).

## Discussion

Jarastatin is a medium-sized disintegrin stabilized by six disulfide bonds. Our findings demonstrate that jarastatin contains all of its hydrophobic residues on the protein surface, yet it remains highly soluble in water. Despite the apparent exposure of these hydrophobic residues, the protein does not oligomerize or aggregate. We conclude that the hydrophobic side chains are shielded from full solvent exposure by clustering with spatially adjacent polar side chains, forming SHCs.

In this study, we aimed to investigate the surface forces of a protein that is extensively cross-linked by disulfide bonds and lacking a canonical hydrophobic core (coreless). To this end, we employed CLEANEX to measure amide protons that exchange with water on a subsecond timescale, termed fast exchangers. We observed a distinct segregation in amide-water exchange behavior: most fast exchangers were located on one face of the protein, corresponding to the region previously identified by our group as the integrin αVβ3 binding site (28). Interestingly, the opposite face was predominantly characterized by slow exchangers and displayed the majority of SHCs. This pattern suggests that surface hydrophobic residues contribute to protein stabilization.

Proteins with high cysteine contents undergo oxidative folding. Sequence analysis *via* disorder prediction algorithms, such as IUPred2A and AIUPred (35, 36), demonstrated that several domains contain disordered regions interspersed with cysteines, with a notable positive correlation between cysteine content and the degree of disorder in the surrounding sequences (37). This tendency toward disorder in disulfide-containing proteins in their reduced state has been observed across various protein domains, including defensin folds, chemokine motifs, zinc finger motifs, carboxypeptidase, three-finger toxin folds, granulin domains, and Kunitz-type protease inhibitor domains (37, 38, 39, 40, 41, 42, 43). In summary, coreless proteins are intrinsically disordered proteins that undergo oxidative folding. Jarastatin is also highly disordered in the reduced state, as predicted by AIUPred (Fig. S6) (36).

Unlike globular proteins, whose folding is driven primarily by hydrophobic collapse to form a hydrophobic core, coreless proteins rely mainly on surface interactions for folding, presumably with SHCs acting as foldons. In a previous publication, our group demonstrated that in the plant defensin Psd2, a coreless protein cross-linked by four disulfide bonds, SHCs contribute to folding stability, suggesting that they function as foldons that facilitate the formation of the native disulfide bond pattern. We also showed that Psd2 SHCs undergo cold denaturation, highlighting the hydrophobic nature of these surface forces (5, 10, 12). Similar behavior was observed for the plant defensin SD5 (4, 44).

Wolfenden and colleagues (45) measured the thermodynamics of transferring molecules mimetic of amino acid side chains between water (W) and cyclohexane (C). These findings indicate that all side chains exhibit hydrophobic characteristics, even polar ones. For example, the transfer of the polar side chain of arginine from an organic solvent to water is enthalpically favorable (ΔH_C-W_ = −13.74 kcal/mol) but entropically unfavorable (−TΔS_C-W_ = 7.86 kcal/mol). The enthalpic term is predominant, making it favorable for this residue to be at protein surfaces, despite the entropic penalty. In contrast, for an apolar side chain such as valine, the transfer to water is enthalpically neutral (ΔH_C-W_ = −1.48 kcal/mol) and entropically unfavorable (−TΔS_C-W_ = 7.04 kcal/mol), making this residue predominantly buried in a hydrophobic core. Notably, both amino acid side chains have similar entropic contributions and behave as hydrophobic moieties. Other polar side chains also show similar behavior. This suggests that the hydrophobic effect observed for Psd2 cold denaturation of the SHC (5) likely arises from interactions between surface-exposed hydrophobic side chains and spatially adjacent polar side chains.

The disulfide bond skeleton in jarastatin contributes to the protein scaffold, shaping the concave form of the binding cleft. A key question remains: do the SHCs mapped in jarastatin also contribute to its oxidative folding and ultimately to its structural conformation? Oxidative protein folding poses a significant challenge due to the vast number of possible disulfide isomers in complex proteins such as jarastatin, which contains six disulfide bonds. Notably, residues that exhibited the highest stability under urea denaturation (Fig. 2*C*) were located around the disulfide bond framework of jarastatin (Fig. S5*A*), suggesting their importance to the fidelity of disulfide bond formation through oxidative folding. Consistent with the stability measurements from urea denaturation, the mapping of the most stable SHCs by monitoring k_ex_ as a function of urea concentration revealed that residues A27, F40, M41, V46, I62, and F71 formed the most urea-resistant clusters (Fig. S5*B*). This behavior indicates that the SHCs, particularly the most stable ones, act as foldons that contribute to oxidative folding. The results of the bis-ANS experiment (Fig. 6) support the idea that the SHCs in jarastatin are resistant to denaturation by urea, maintaining their structure even at concentrations up to 8 M. This experiment revealed organized, surface-exposed hydrophobic regions in jarastatin that persist under these conditions.

While oxidation is essential for oxidative protein folding, reduction is equally important for preventing nonnative disulfide formation and for maintaining the endoplasmic reticulum redox homeostasis (46). This reduction is mediated by the glutathione and thioredoxin systems (47). A balance of oxidation, reduction, and efficient disulfide isomerization allows for the necessary reshuffling to achieve a minimally frustrated state. Foldons play a critical role here by promoting the lowest-energy conformation through interactions within the SHCs. The large number of protein disulfide isomerase (PDI) isoforms in higher eukaryotes likely reflects their distinct substrate specificities, with their disulfide isomerization and chaperone activities facilitating the folding, unfolding, and reshuffling of specific regions in cysteine-containing proteins (47).

The residues with the highest local stability colocalized with most of the SHCs, forming the protein scaffold, whereas residues with the lowest local stability aligned with the jarastatin binding site for integrin αVβ3 (28), as noted earlier. The binding cleft has a concave shape that complements the convex loop (211-RTAQAIF-217) of the αV integrin subunit. This cleft is composed of surface-exposed polar and charged residues that interact with integrins primarily through salt bridges and hydrogen bonds. The combination of the concave shape, the predominance of polar and charged residues in the binding cleft, and the concentration of SHCs on the opposite hydrophobic face, which defines the molecular shape, is critical for integrin binding and the biological activity of jarastatin.

Most of the residues mapped as participating in the interaction with the integrin through NMR-derived data, such as chemical shift perturbation, line broadening and changes in ^15^N-R_2_ (28), are fast exchangers. Interestingly, they include residues directly involved in binding or in the vicinity (Table S3). This behavior has been observed earlier for the lectin galectin (48), where it was observed in a CLEANEX experiment with 13 fast exchangers, most of which participate directly in binding to the glycan. There are two reasons to explain this phenomenon. One of them is that the amide of fast exchangers is more prone to interact *via* hydrogen bonding to a molecular partner. The second reason is that the desolvation energy of these regions, which are more permeable to water, is lower. Desolvation of the binding site is an important step in any molecular recognition. The more easily desolvation occurs, the more prone the site is to interaction (49).

In summary, in this study, we used CLEANEX to assess the stability of the SHCs in jarastatin. These clusters are remarkably stable, resisting denaturation even in up to 8 M urea. The most stable SHCs function as protein scaffolds, shaping their concave structure. In jarastatin, SHCs act as foldons, guiding oxidative folding and contributing to the formation of the disulfide bond framework, which is essential for establishing the concave shape and, ultimately, the binding cleft. The residues forming the binding cleft are predominantly fast exchangers, enhancing the binding propensity through their amide groups, which are available for hydrogen bonding, and the relatively low desolvation energy of regions that are more permeable to water.

## Experimental procedures

### Jarastatin expression and purification

The expression and purification of jarastatin were conducted according to previous methods (28, 29). The jarastatin gene/cDNA was inserted into the pPIC9 vector, and peptide expression in *Komagataella phaffii* was induced by the addition of methanol. ^15^N-labeled jarastatin was secreted into the supernatant.

The purification of ^15^N-jarastatin was performed *via* a Superdex 75 size exclusion column coupled to an ÄKTA Pure system (GE Healthcare). The column was equilibrated with 5 mM sodium phosphate buffer (pH 6.0) at a constant flow rate of 1 ml/min and loaded with 15 ml of medium containing ^15^N-jarastatin. Elution was performed with the same buffer, and the absorbance was monitored at 280 nm. The fractionated peaks were subjected to 18% SDS‒PAGE to identify the target protein and assess its purity (adapted from Vasconcelos *et al.*, 2022, (29)). The NMR samples contained 5 mM sodium phosphate buffer at pH values of 6.0, 6,5, 7.0, or 7.5. The jarastatin concentration was 191 μM.

### Fitting of the urea denaturation profiles

We used a series of ^15^N-^1^H-HSQC in several urea concentrations. We calculated the chemical shift perturbation (CSP) *via* the following equation:

$$CSP=\sqrt{{{\Delta \delta }_{1H}}^{2}+{\left(\frac{{\Delta \delta }_{15N}}{6.6}\right)}^{2}}$$

Where Δδ is the chemical shift differences for ^1^H and ^15^N.

The fitting of the CSP as a function of the urea concentration (Fig. S4) was performed considering a two-state equilibrium between a folded (F) and an unfolded (U) state. The unfolding free energy for each concentration of urea (ΔG([urea])) follows the equations (30) below:

(equation 1) $$\Delta G\left(\left[urea\right]\right)=-RT\;\ln\left[\frac{f_{U}}{f_{F}}\right]=-RT\;\ln\left(\frac{\theta }{1-\theta }\right)$$

where $f_{U}$ and $f_{F}$ are the molar fractions of U and F, respectively. R is the universal gas constant, and T is the absolute temperature (298 K). The molar fractions were obtained from the urea denaturation profiles followed by the CSP.

The unfolding free energy in the absence of urea (ΔG_F-U_) was obtained according to the following equation:

(2) $${\Delta G}_{F-U}=\Delta G\left(\left[urea\right]\right)-m\left[urea\right]$$

By combining equations (equation 1), (2), we derived the following equation, which was used to fit the urea denaturation profiles and obtain the values of ΔG_F-U_ and m. The equations were implemented in OriginPro 2021 (OriginLab Corporatio), followed by nonlinear fitting.

(3) $$CSP=\left({CSP}_{\max}-CSP\right)e^{\left(\frac{m\left[urea\right]}{RT}-\frac{{\Delta G}_{F-U}}{RT}\right)}+{CSP}_{o}$$

where ${CSP}_{\max}$, m and ${\Delta G}_{F-U}$ are adjustable parameters, while ${CSP}_{o}$ is fixed at zero.

### CLEANEX and fitting of water exchange rate (k_ex_)

To measure the water exchange rate (k_ex_) of HN, we run the NMR experiment CLEANEX (13) on a Bruker Avance III HD 700 MHz instrument using the version enclosed in the Bruker pulse sequence library (fhsqccxf3gpph). The following CLEANEX‒PM spin‒locking mixing times were used: 5, 10, 20, 40, 80, and 120 ms. The reference experiment was a fast HSQC (fhsqcgpph). The exchange rates were quantified by fitting the cross-peak volumes as a function of time to a single exponential decay. The spin-locking field in CLEANEX-PM was 4.8 kHz, the number of complex points was 1024 and 100 in the t2 and t1 dimensions, and the spectral widths were 9803.922 and 2129.107 Hz in the t2 and t1 dimensions, respectively. The acquisition times were 52.2224 and 46.9668 ms in the t2 and t1 dimensions, respectively. The number of transients was set to 64, and the recycle delay for each scan was 2 s.

The cross-peak intensity (*I*) was obtained from the CLEANEX experiments, and *I*_*ref*_ was obtained from the reference ^1^H-^15^ N fast HSQC spectrum.

(4) $$\frac{I}{I_{ref}}=\left(\frac{k_{ex}}{R_{1A}+k_{ex}-R_{1B}}\right)\left(e^{{-R}_{1B}\tau_{mix})}-e^{-\left(R_{1A}+k_{ex}\right)\tau_{mix})}\right)$$

k_ex_ is the rate of the amide water exchange forward rate constant (HN to H_2_0), R_1A_ is the apparent relaxation rate, which is a combination of longitudinal and relaxation constants, and R_1B_ is the apparent water relaxation rate, which is 0.45 Hz. where τ_mix_ is the CLEANEX-PM mixing time. We did not correct for the water saturation. The equations were implemented in OriginPro 2021 (OriginLab Corporation), followed by nonlinear fitting.

The experimental error of $\frac{I}{I_{ref}}$ was obtained from the signal to noise ratio of the CLEANEX spectra according to the following equation:

(5) $$\Delta_{\frac{I}{I_{ref}}}=\frac{I}{I_{ref}}\;\sqrt{{\left(\frac{SD}{I_{ref}}\right)}^{2}+{\left(\frac{SD}{I}\right)}^{2}}$$

Where $\Delta_{\frac{I}{I_{ref}}}$ is the experimental error and SD is the standard deviation of the spectral noise. SD did not vary significantly from the different CLEANEX spectra. We got SD from the spectral at 50 ms of mixing time. The kex experimental error was propagated during the fitting according to equation using direct weighting in the software OriginPro 2021 (OriginLab Corporation).

### Water exchange protection factor

Water exchange protection factor (P) was calculated from the observed kex for each pH and the intrinsic amide water exchange rate constant (*kint*) was obtained from the spreadsheets obtained from Walter Englander laboratory (https://hx2.med.upenn.edu/) (50, 51, 52).

(6) $$P=\frac{k_{\mathrm{int}}}{k_{ex}}$$

### Dipolar interaction of the protein amide hydrogen with water and urea

NOESY-HSQC (53) and ROESY-HSQC (54) spectra were used to measure the nuclear Overhauser effect (NOE) in the laboratory frame and in the rotating frame (ROE), respectively, between the amide hydrogens of jarastatin and water and urea. The sample contained 191 μM jarastatin in 5 mM sodium phosphate buffer at pH 6.0 with 2 M urea. Gradient selection and sensitivity enhancement were applied in the spectra acquisition (55).

The data were acquired with 1024, 80, and 140 complex points in the F3 (^1^H), F2 (^15^N), and F1 (^1^H) dimensions, respectively, using 512 scans and a mixing time of 20 ms. The spectra were sampled nonuniformly (NUS) *via* multidimensional Poisson‒Gap scheduling (56), with 190 complex points for the NOESY‒HSQC and 140 for the ROESY‒HSQC. Spectral processing was performed *via* nmrPipe (57), which employs the NESTA algorithm for multidimensional NUS reconstruction (58).

### Spectroscopic measurements—Bis-ANS

Fluorescence emission spectra were recorded *via* a spectrofluorometer (Cary Eclipse Agilent Technologies). The fluorescence emission of the hydrophobic probe 4,4′-dianilino-1,1′-binaphthyl-5,5′-disulfonic acid (bis-ANS, purchased from Molecular Probes) was recorded from 400 to 700 nm, with excitation at 360 nm, using excitation and emission slits of 10 nm. For the assay, a sample of jarastatin at a concentration of 5 μM in 5 mM sodium phosphate buffer, pH 6.0, containing bis-ANS (1:1 ratio of jarastatin), was titrated with urea solutions of 0, 1, 2, 3, 4, 5, 6, 7, and 8 M. A control experiment without jarastatin was also conducted. The difference spectra between the experiments with and without jarastatin were obtained for each urea concentration.

## Data availability

All data reported in this paper will be shared by the corresponding author (falmeida@bioqmed.ufrj.br).

## Supporting information

This article contains the following figures and tables as supporting information: Figs. S1–S6 and Tables S1–S3 (18, 28).

## Conflict of interest

The authors declare that they have no conflicts of interest with the contents of this article.
