# Supplementary material for: Surface hydrophobic clusters modulate the folding stability and molecular recognition of the disintegrin jarastatin
Source: J Biol Chem. 2025 Feb 11;301(3):108294. doi: 10.1016/j.jbc.2025.108294 (PMC11930076; doi:10.1016/j.jbc.2025.108294)
Supplement: Supporting information [file mmc1.pdf]

Supporting Information

Surface hydrophobic clusters modulate the folding stability and molecular recognition of the disintegrin Jarastatin.

Ariana A. Vasconcelos[1,2*], Russolina B. Zingali[1], Fabio C. L. Almeida[1,2*].

[1]Institute of Medical Biochemistry Leopoldo de Meis (IBqM), Federal University of Rio de Janeiro (UFRJ), Brazil.

[2]National Center for Structural Biology and Bioimaging (CENABIO), Federal University of Rio de Janeiro (UFRJ), Brazil.

*Correspondent authors

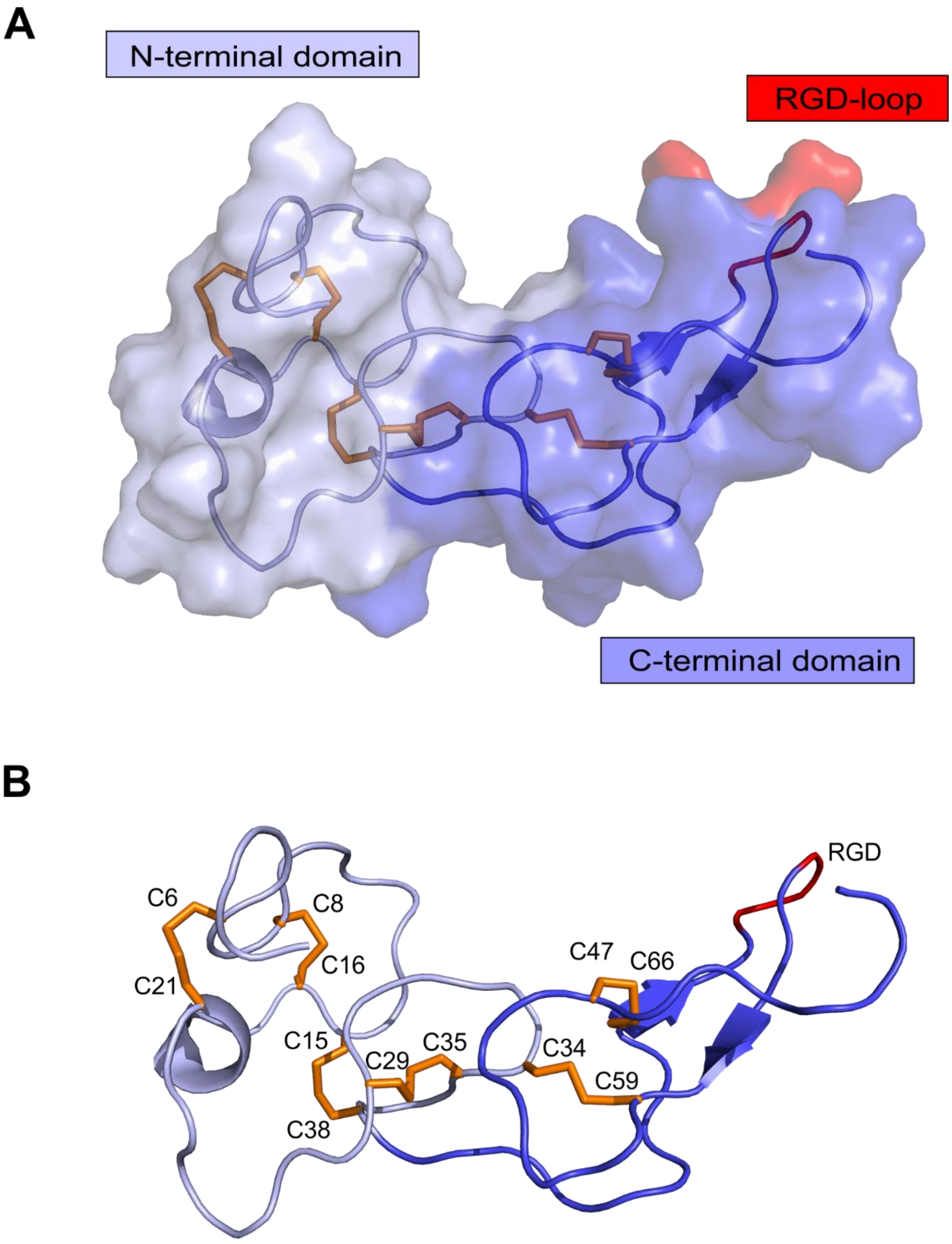

Figure S1. Structure of jarastatin. (A) Ribbon/Surface representation of jarastatin showing the N-terminal domain (1-38) in light blue, the C-terminal domain in blue and the RGD-loop in red. (B) Ribbon representation of jarastatin showing the disulfide bonds in orange and the RGD-loop in red.

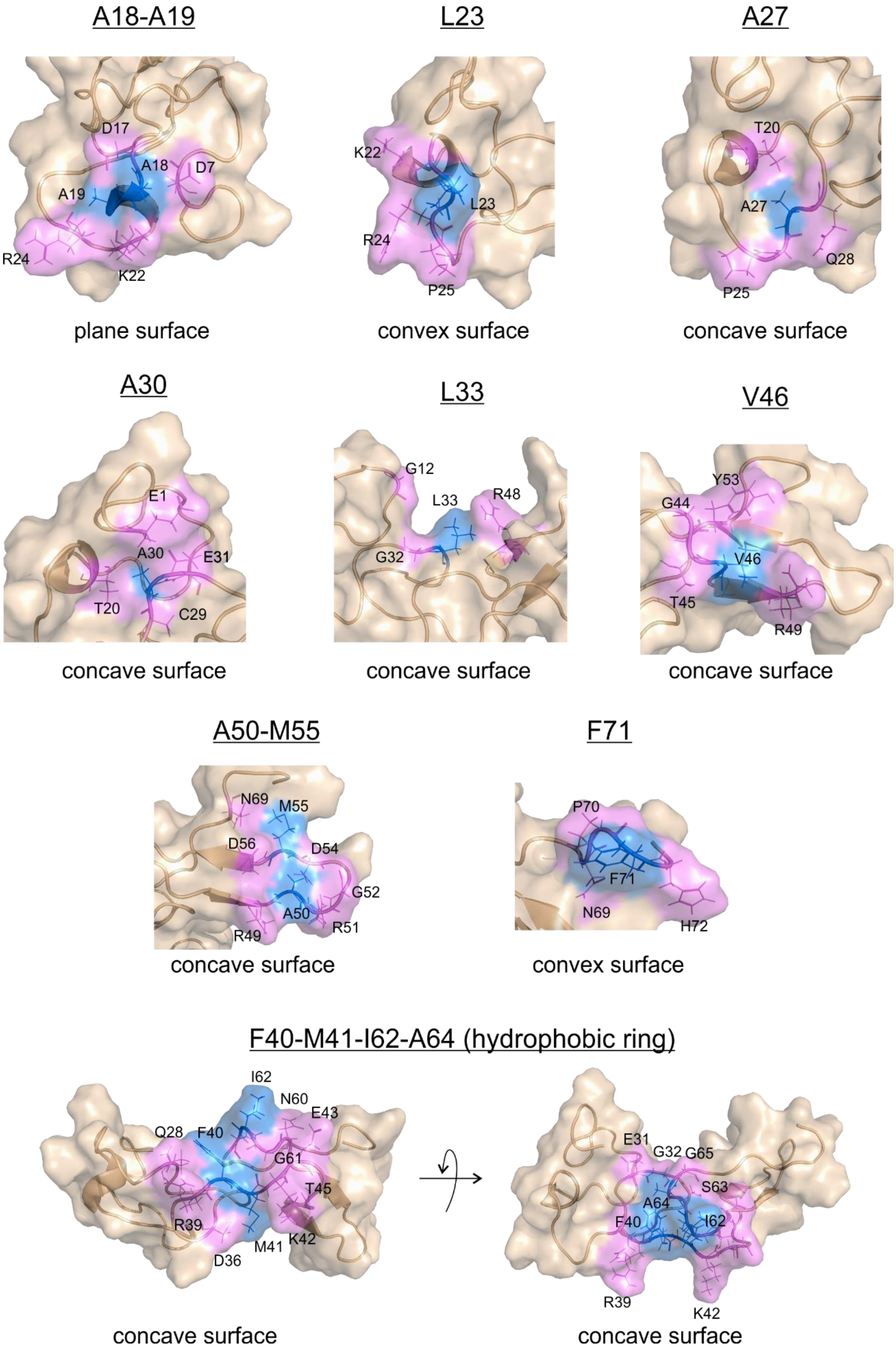

Figure S2. Ribbon and surface representation of each surface hydrophobic cluster of jarastatin, highlighting the exposed hydrophobic residues in blue and the spatially adjacent residues in violet.

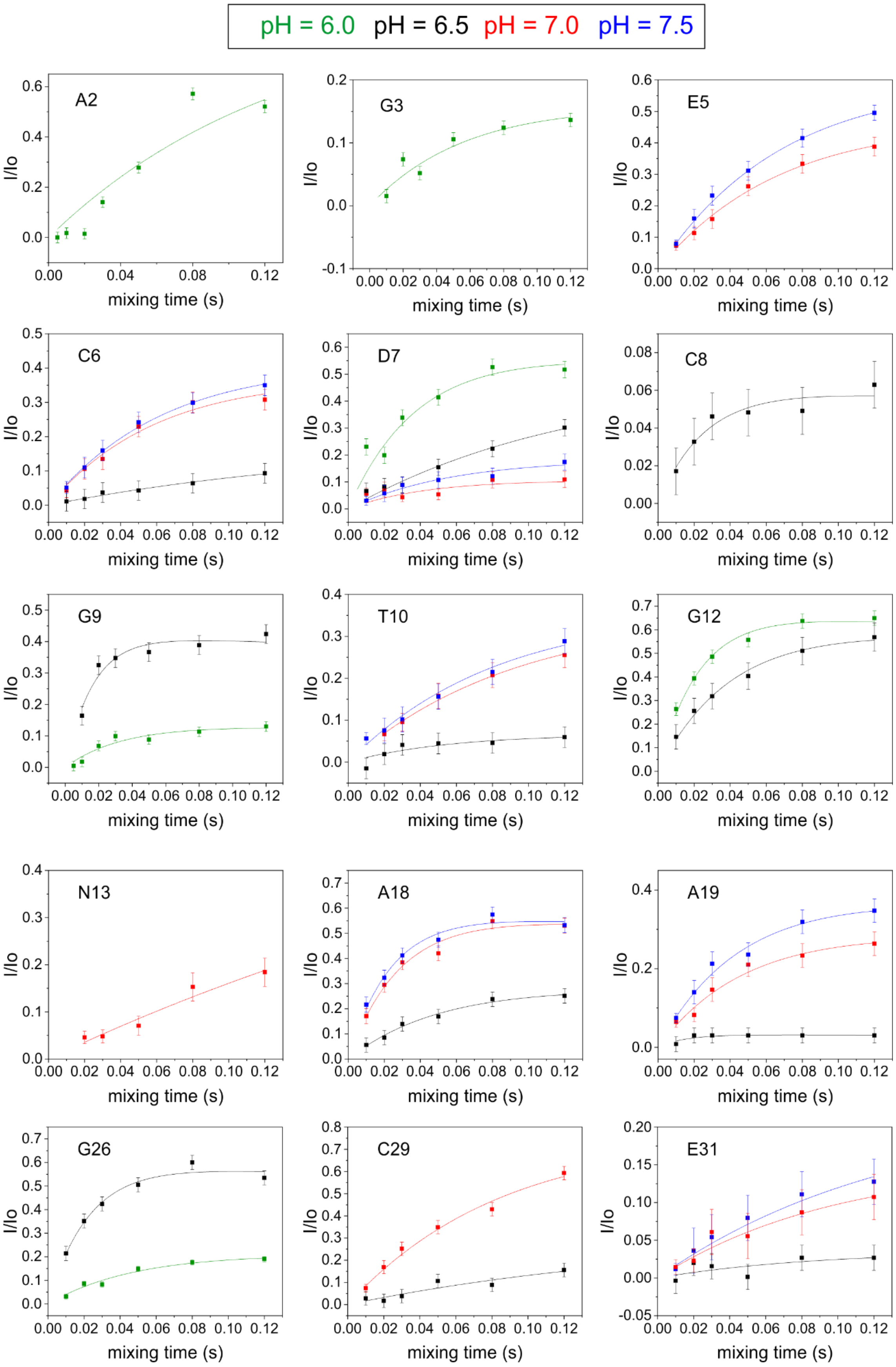
pH = 6.0 pH = 6.5 pH = 7.0 pH = 7.5
A2
G3
E5
C6
D7
C8
G9
T10
G12
N13
A18
A19
G26
C29
E31
I/Io
mixing time (s)

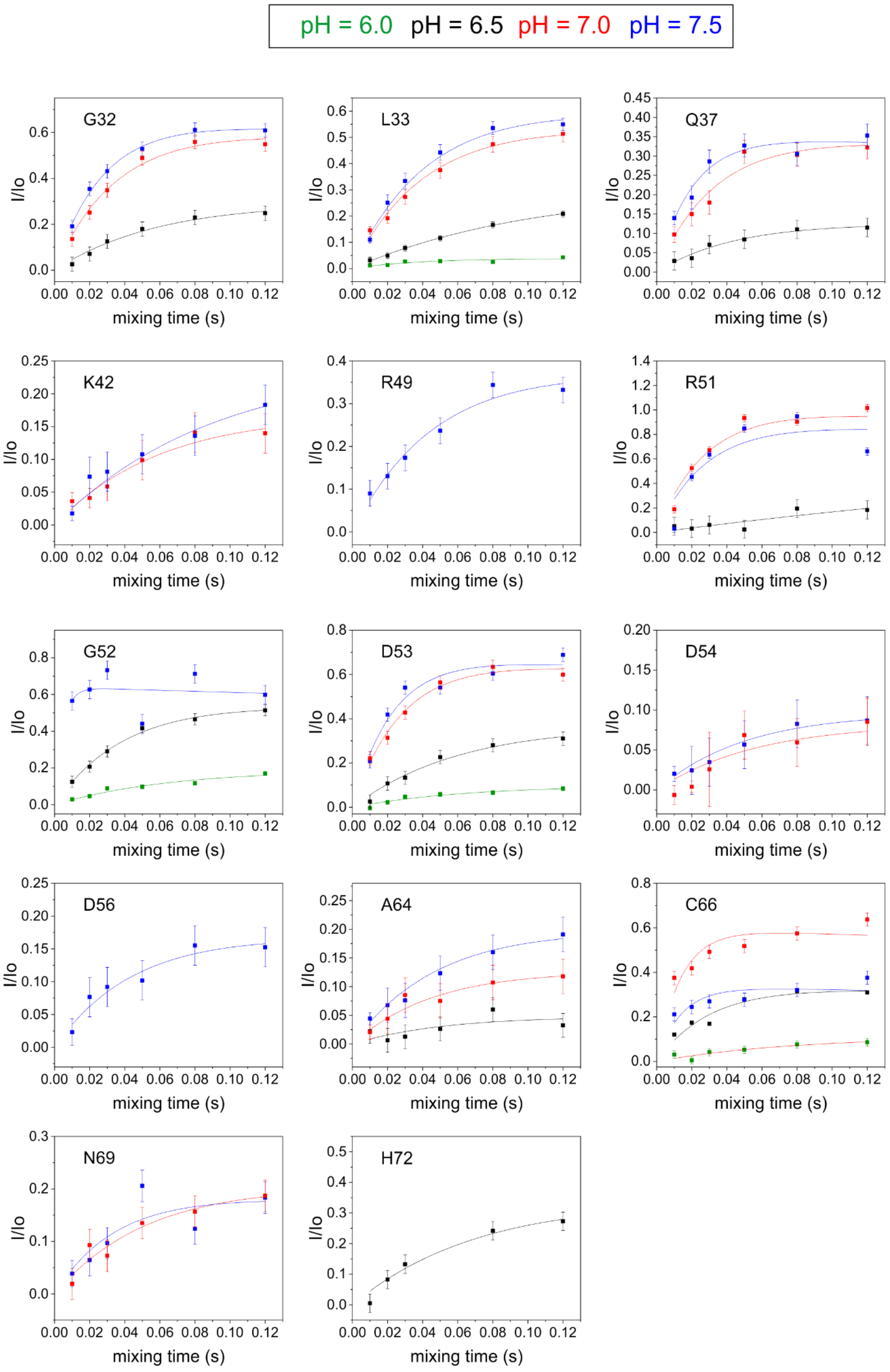
pH = 6.0
pH = 6.5
pH = 7.0
pH = 7.5
G32
L33
Q37
K42
R49
R51
G52
D53
D54
D56
A64
C66
N69
H72
I/Io
mixing time (s)

Figure S3. CLEANEX profile of amide protons of jarastatin in pH 6.0 (green), 6.5 (black), 7.0 (red), and 7.5 (blue). The fitting of CLEANEX data was performed using Equation 4, according to the methods section.

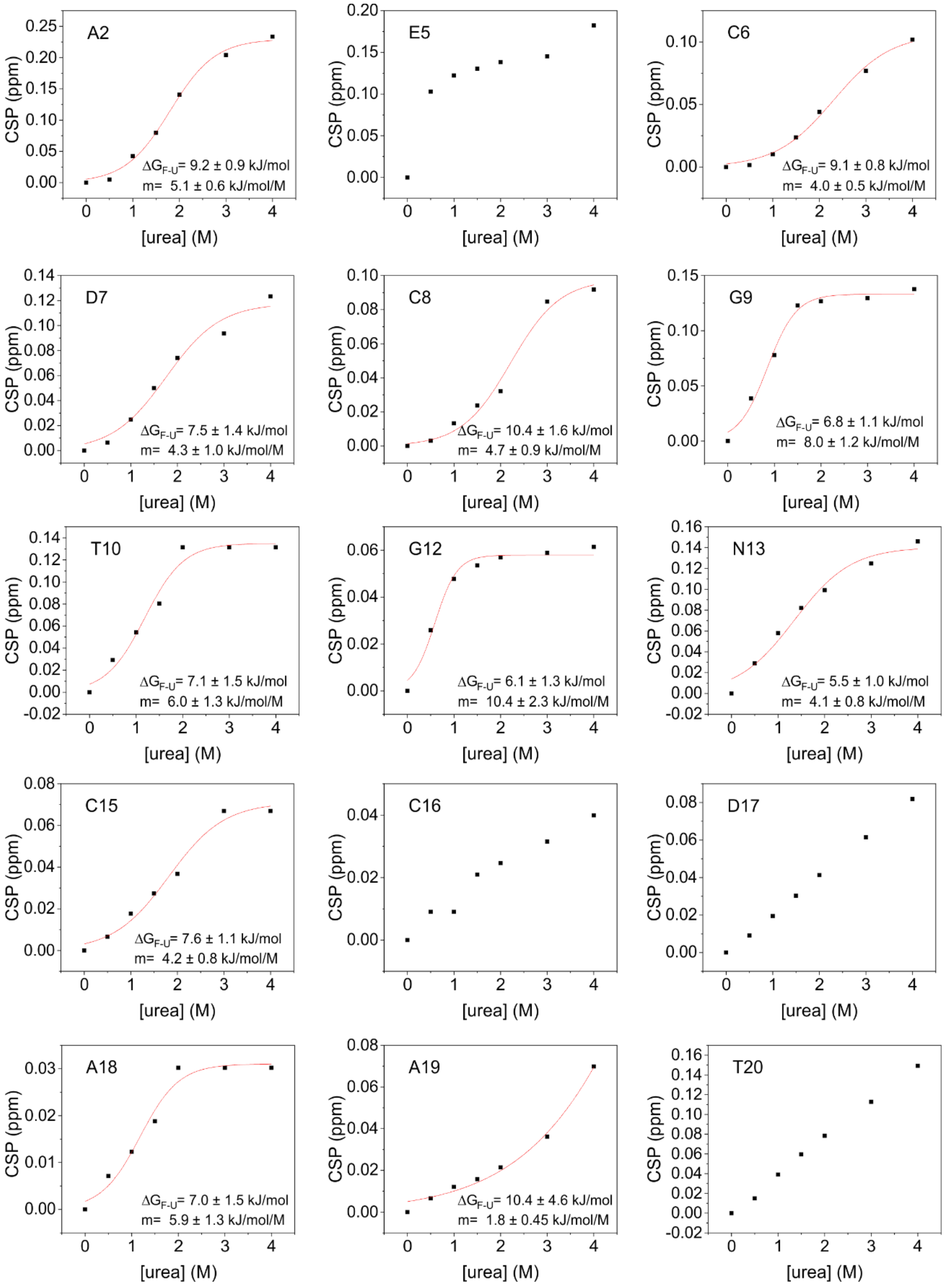

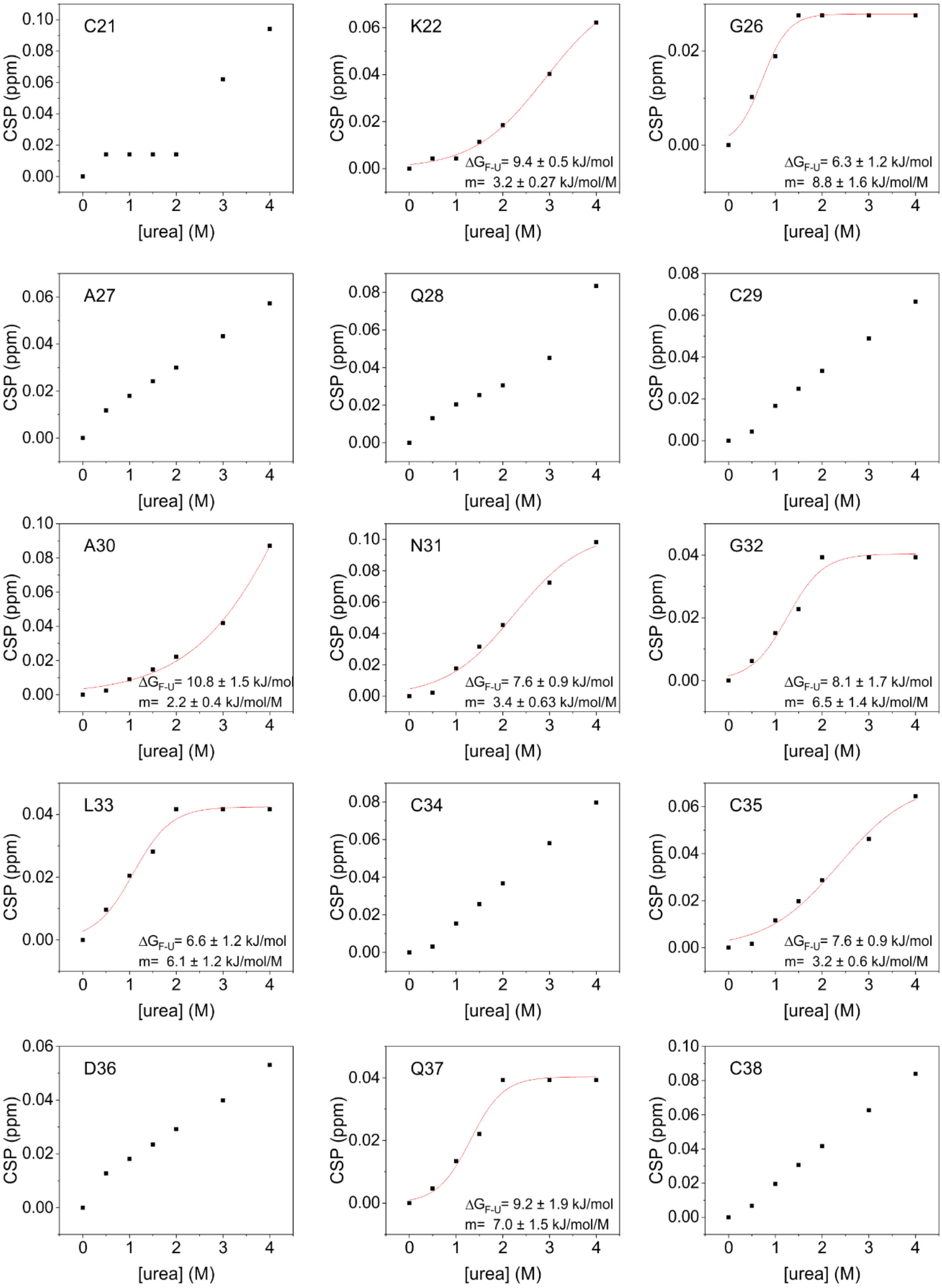

C21
K22
ΔG_F-U= 9.4 ± 0.5 kJ/mol
m= 3.2 ± 0.27 kJ/mol/M
G26
ΔG_F-U= 6.3 ± 1.2 kJ/mol
m= 8.8 ± 1.6 kJ/mol/M
A27
Q28
C29
A30
ΔG_F-U= 10.8 ± 1.5 kJ/mol
m= 2.2 ± 0.4 kJ/mol/M
N31
ΔG_F-U= 7.6 ± 0.9 kJ/mol
m= 3.4 ± 0.63 kJ/mol/M
G32
ΔG_F-U= 8.1 ± 1.7 kJ/mol
m= 6.5 ± 1.4 kJ/mol/M
L33
ΔG_F-U= 6.6 ± 1.2 kJ/mol
m= 6.1 ± 1.2 kJ/mol/M
C34
C35
ΔG_F-U= 7.6 ± 0.9 kJ/mol
m= 3.2 ± 0.6 kJ/mol/M
D36
Q37
ΔG_F-U= 9.2 ± 1.9 kJ/mol
m= 7.0 ± 1.5 kJ/mol/M
C38
CSP (ppm)
[urea] (M)

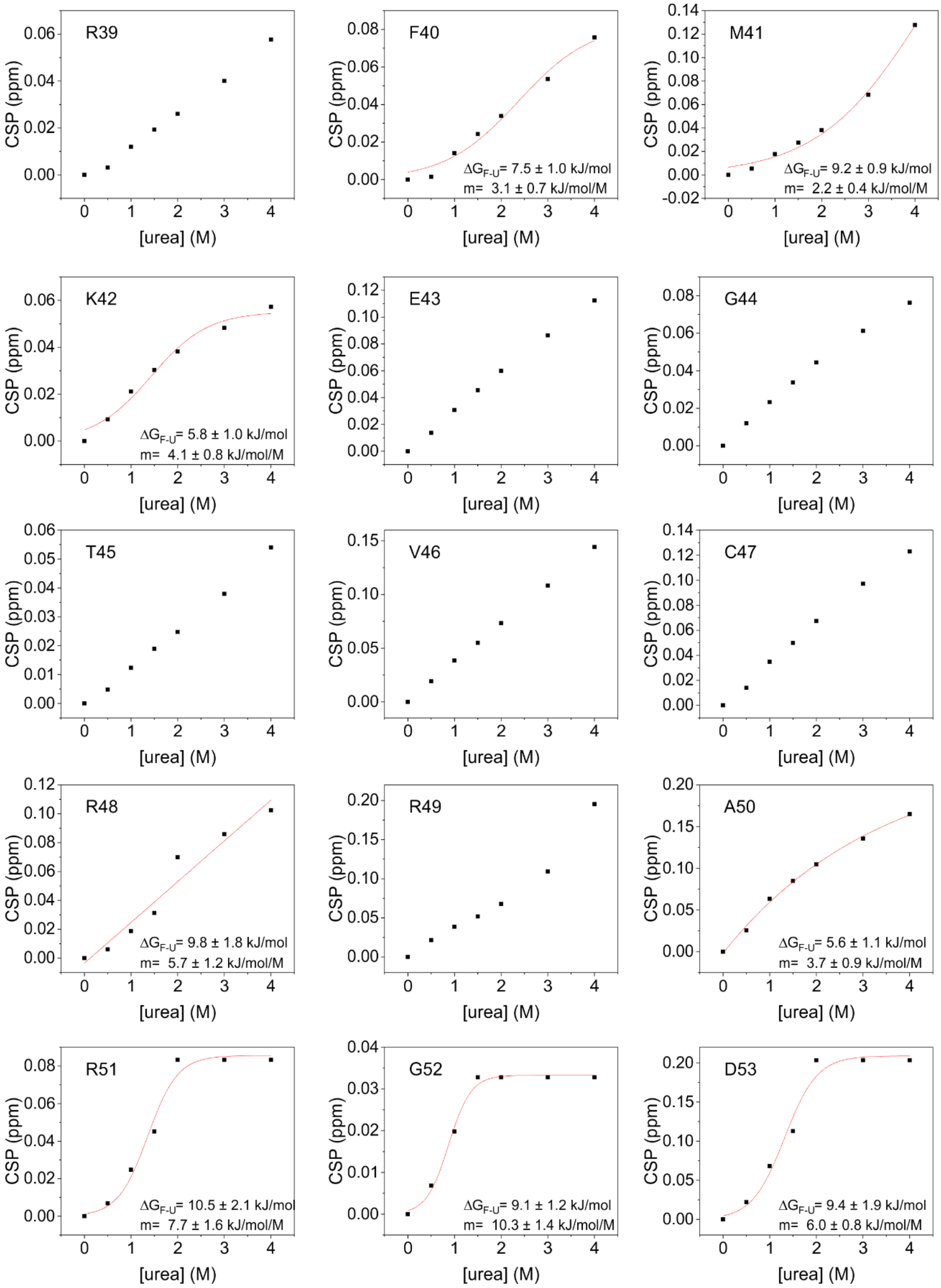
R39
CSP (ppm)
[urea] (M)
F40
ΔG_F-U = 7.5 ± 1.0 kJ/mol
m= 3.1 ± 0.7 kJ/mol/M
M41
ΔG_F-U = 9.2 ± 0.9 kJ/mol
m= 2.2 ± 0.4 kJ/mol/M
K42
ΔG_F-U = 5.8 ± 1.0 kJ/mol
m= 4.1 ± 0.8 kJ/mol/M
E43
G44
T45
V46
C47
R48
ΔG_F-U = 9.8 ± 1.8 kJ/mol
m= 5.7 ± 1.2 kJ/mol/M
R49
A50
ΔG_F-U = 5.6 ± 1.1 kJ/mol
m= 3.7 ± 0.9 kJ/mol/M
R51
ΔG_F-U = 10.5 ± 2.1 kJ/mol
m= 7.7 ± 1.6 kJ/mol/M
G52
ΔG_F-U = 9.1 ± 1.2 kJ/mol
m= 10.3 ± 1.4 kJ/mol/M
D53
ΔG_F-U = 9.4 ± 1.9 kJ/mol
m= 6.0 ± 0.8 kJ/mol/M

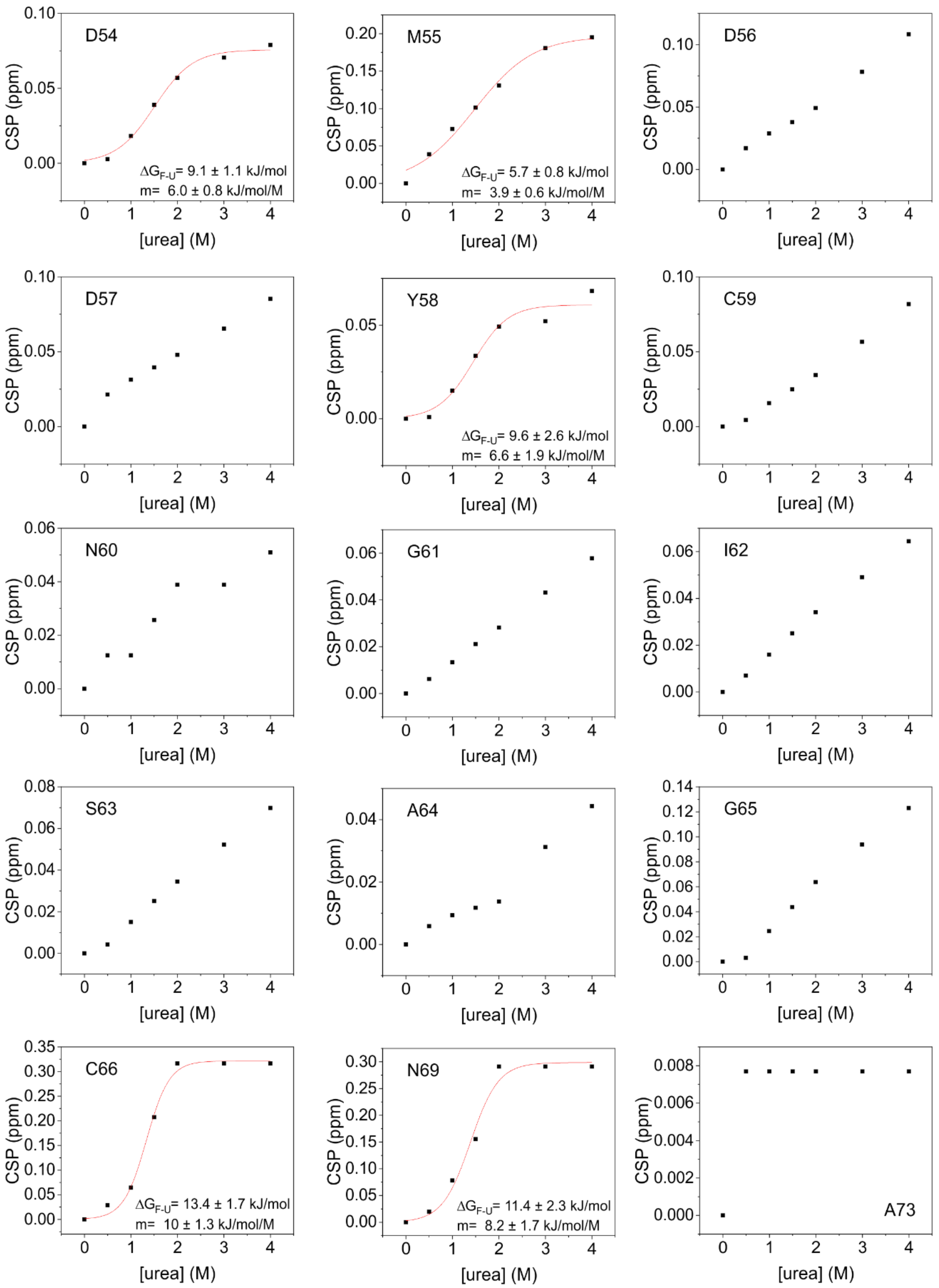

Figure S4. Chemical shift perturbation of the jarastatin amide protons as a function of urea concentration.

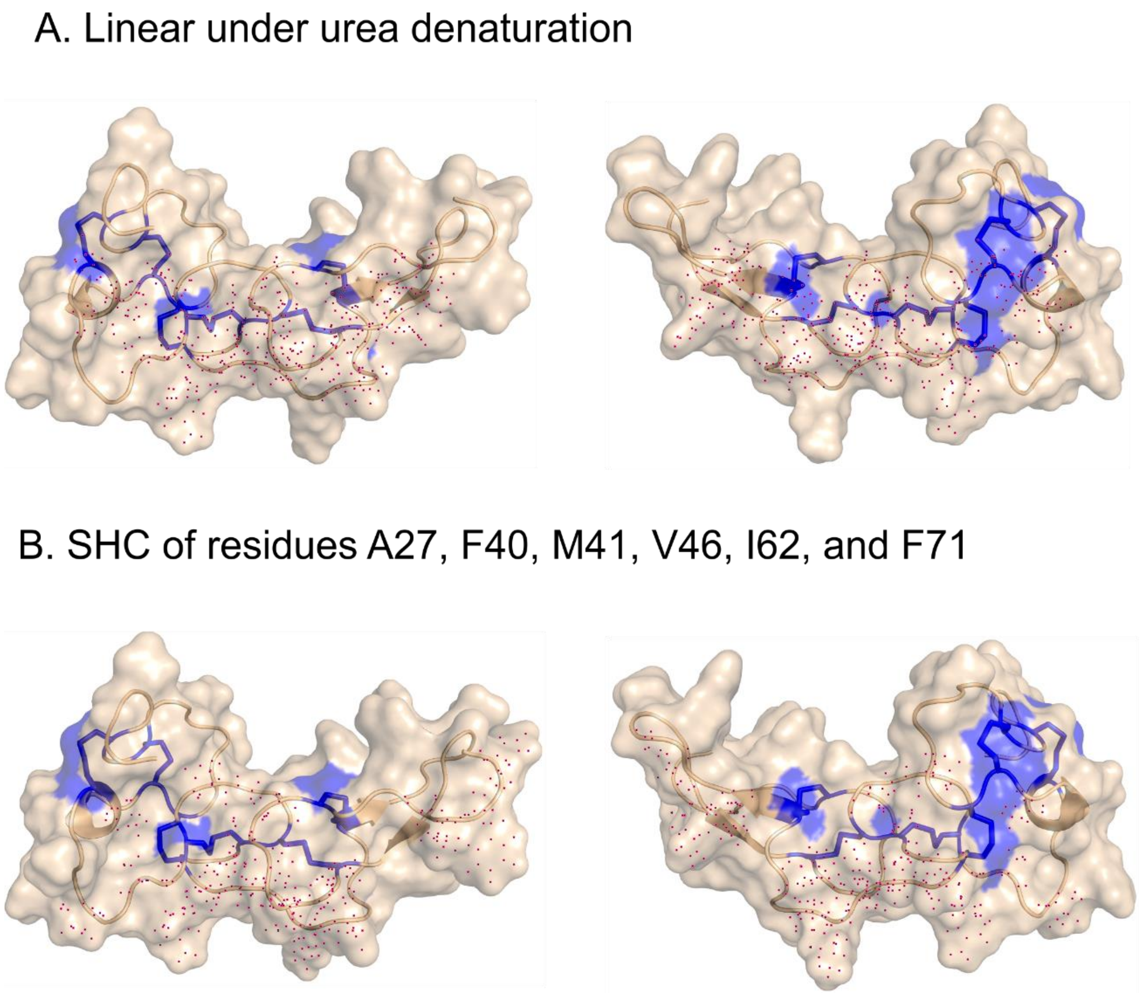

Figure S5. Ribbon and surface representation of jarastatin. The disulfide bonds are in blue, forming a disulfide skeleton that contributes to the concave shape of the protein. (A) The red dots show the atoms of the residues that displayed a linear profile of the CSP upon urea denaturation. These are the most stable regions of the protein and surround the disulfide skeleton. (B) The red dots show the atoms of the residues that compose the amides that remains protected to water exchange even at the highest urea concentration. These are the most stable hydrophobic surface clusters, which also surround the disulfide skeleton.

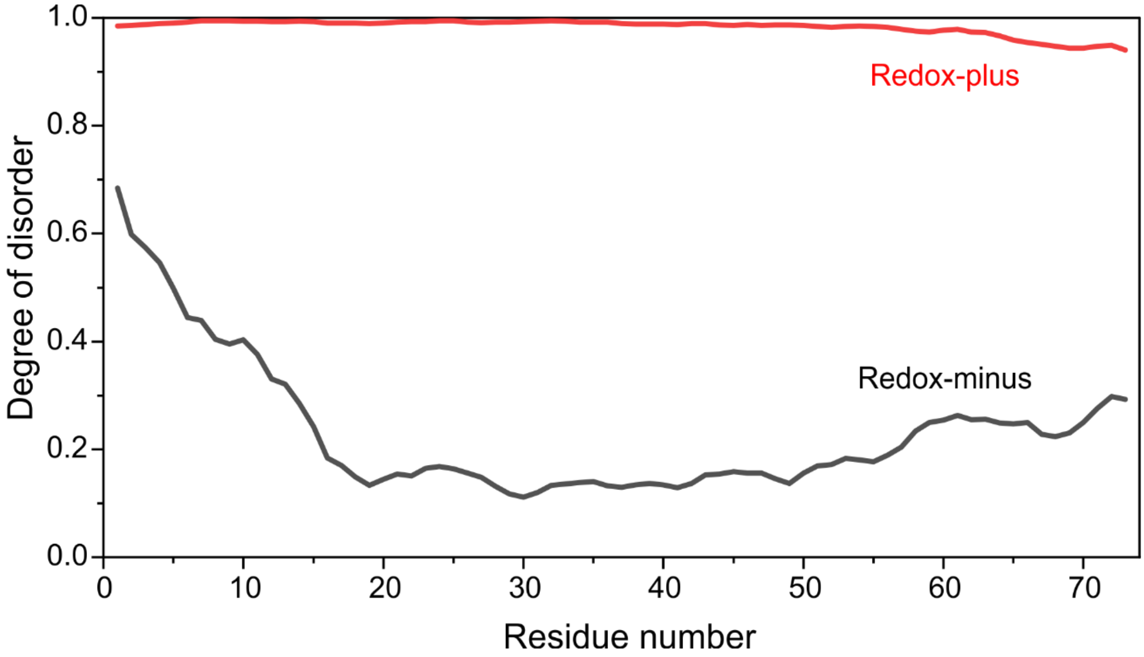

Figure S6. Prediction of the structure disorder of jarastatin in the oxidized (redox-minus) and reduced (redox-plus) states using AIUPRED software (https://aiupred.elte.hu/) (19). In the reduced state jarastatin is an intrinsically disordered protein and becomes ordered upon disulfide bond formation under oxidative folding.

Table S1. Water exchange rates of the amide protons of jarastatin detectable by CLEANEX ($k_{ex} > 1$ s^{-1}) as a function of urea concentration at pH 6.0. The first column describes our interpretation of the observed behavior of each amide. We classified as exposed even in the absence of urea, becomes exposed upon addition of urea, and protected from water exchange even at the highest urea concentration. We also show the prolines and the residues that were not assigned.

Interpreted Behavior	Residue	k_{ex}, s^{-1} (pH 6.0)						
		0 M	0.5 M urea	1 M urea	1.5 M urea	2.0 M urea	3.0 M urea	4.0 M urea
**Exposed**	A2	7.2 ± 1.0			3.5 ± 1.2	12 ± 1.1	41 ± 9.6	41 ± 12
**Exposed**	G3	2.8 ± 1.0	10 ± 1.0	13 ± 1.2				
**Becomes exposed**	E4				7.5 ± 3.0	15 ± 1.1	26 ± 8.9	74 ± 28
**Becomes exposed**	E5				6.6 ± 0.5	16 ± 3.2	49 ± 14	116 ± 36
**Becomes exposed**	C6			5.9 ± 1.6	5.4 ± 0.4	15 ± 3.1	50 ± 7.9	29 ± 6.1
**Exposed**	D7	15 ± 4.0	1.0 ± 0.4	2 ± 1.5	4.7 ± 0.6	14 ± 1.8	24 ± 5.1	19 ± 4.5
**Protected**	C8							
**Exposed**	G9	3.9 ± 0.9	12 ± 1.6	14 ± 3.1				
**Protected**	T10							
**Proline**	P11							
**Exposed**	G12	27 ± 4.6	6.7 ± 3.2					
**Becomes exposed**	N13			1.4 ± 0.6	5.2 ± 0.7	14 ± 0.6	34 ± 6.9	31 ± 3.5
**Proline**	P14							
**Protected**	C15							
**Becomes exposed**	C16				1.8 ± 0.4	12 ± 1.8	13 ± 2.0	10 ± 2.2
**Protected**	D17							

**Becomes exposed**	A18		4.8 ± 1.3	10 ± 1.0	5.8 ± 1.7			
**Becomes exposed**	A19			4.3 ± 0.7	6.8 ± 0.6	13 ± 3.7	48 ± 22	56 ± 13
**Protected**	T20							
**Becomes exposed**	C21			2.4 ± 1.1	6.2 ± 1.1	6.8 ± 0.3	1.7 ± 0.5	
**Protected**	K22							
**Not assigned**	L23							
**Not assigned**	R24							
**Proline**	P25							
**Exposed**	G26	4.1 ± 1.0	10 ± 0.7	11 ± 3.5				
**Protected**	A27							
**Protected**	Q28							
**Protected**	C29							
**Becomes exposed**	A30				1.3 ± 0.5	9.2 ± 2.5	9.0 ± 1.8	5.3 ± 1.1
**Becomes exposed**	E31				3.2 ± 0.7	11 ± 0.9	16 ± 1.4	17 ± 1.3
**Becomes exposed**	G32		4.9 ± 0.8	12 ± 1.5	7.6 ± 0.3			
**Exposed**	L33	0.86 ± 0.38	3.4 ± 0.9	9.4 ± 1.8	6.8 ± 0.8	13.3 ± 4.8	48 ± 17	
**Protected**	C34							
**Protected**	C35							
**Protected**	D36							
**Becomes exposed**	Q37		3.4 ± 1.5	7.7 ± 0.4	5.7 ± 0.8			
**Protected**	C38							
**Protected**	R39							
**Protected**	F40							

**Protected**	M41							
**Becomes exposed**	K42			2.0 ± 0.5	4.4 ± 0.6	13 ± 1.0	24 ± 2.7	21 ± 0.9
**Becomes exposed**	E43					2.6 ± 0.8	2.8 ± 0.1	2.9 ± 0.6
**Protected**	G44							
**Protected**	T45							
**Becomes exposed**	V46					4.0 ± 0.9	6.7 ± 1.5	3.3 ± 0.8
**Protected**	C47							
**Protected**	R48							
**Becomes exposed**	R49			8.1 ± 2.1	6.4 ± 0.4	16 ± 2.4	33 ± 18	31 ± 12
**Becomes exposed**	A50					4.8 ± 1.1	4.8 ± 1.0	3.7 ± 0.9
**Becomes exposed**	R51			4.8 ± 1.7	8.7 ± 1.4	8.0 ± 4.0	6.5 ± 0.6	
**Exposed**	G52	2.7 ± 0.5	7.2 ± 0.8	18 ± 2.1	1.0 ± 0.1	5.5 ± 0.6		
**Exposed**	D53	1.5 ± 0.4	6.7 ± 1.3	16 ± 1.0	7.8 ± 3.0			
**Becomes exposed**	D54				1.9 ± 0.8			4.4 ± 0.6
**Protected**	M55							
**Becomes exposed**	D56							1.4 ± 0.5
**Protected**	D57							
**Protected**	Y58							
**Protected**	C59							
**Becomes exposed**	N60					12 ± 3.1	16 ± 5.6	8.5 ± 2.2
**Protected**	G61							

**Protected**	I62							
**Protected**	S63							
**Becomes exposed**	A64			2.3 ± 0.7	5.9 ± 1.1	13 ± 2.3	17 ± 1.0	12 ± 2.1
**Becomes exposed**	G65					3.6 ± 0.9	4.0 ± 0.9	3.1 ± 0.5
**Exposed**	C66	1.4 ± 0.4	5.3 ± 1.6	18 ± 0.8			9.8 ± 3.2	
**Proline**	P67							
**Protected**	R68							
**Becomes exposed**	N69			3.8 ± 1.2				
**Proline**	P70							
**Protected**	F71							
**Protected**	H72							
**Protected**	A73							

Table S2. Water exchange rates of the amide protons of jarastatin detectable by CLEANEX ($k_{ex} > 1$ s^{-1}) as a function of urea concentration at pH 7.5. The first column describes our interpretation of the observed behavior of each amide. We classified as exposed even in the absence of urea, becomes exposed upon addition of urea, and protected from water exchange even at the highest urea concentration. We also show the prolines and the residues that were not assigned.

Interpreted Behavior	res number	kex				
		pH 7.5	pH 7.5	pH 7.5	pH 7.5	pH 7.5
		0 M	1M urea	2.0M urea	3.0M urea	4.0M urea
**Exposed/undetectable**	A2					
**Exposed/undetectable**	G3					
**Exposed/undetectable**	E4					
**Exposed**	E5	9.3 ± 0.3	9.5 ± 0.9			
**Exposed**	C6	6.7 ± 0.2	9.9 ± 1.4			
**Exposed**	D7	4.5 ± 0.5				
**Becomes exposed**	C8			6.0 ± 0.6		10 ± 3.0
**Exposed/undetectable**	G9					
**Exposed**	T10	3.9 ± 0.2	12 ± 3.2		31 ± 4.5	
**Proline**	P11					
**Exposed/undetectable**	G12					
**Exposed/undetectable**	N13					
**Proline**	P14					
**Protected**	C15					
**Exposed**	C16	2.8 ± 0.4			21 ± 1.0	34 ± 12

**Protected**	D17					
**Exposed**	A18	24 ± 1.6				
**Exposed**	A19	9.0 ± 0.8	11 ± 1.3			
**Becomes exposed**	T20			5.0 ± 1.1		
**Exposed/undetectable**	C21					
**Protected**	K22					
**Not assigned**	L23					
**Not assigned**	R24					
**Proline**	P25					
**Exposed/undetectable**	G26					
**Protected**	A27					
**Protected**	Q28					
**Protected**	C29					
**Becomes exposed**	A30				22 ± 6.0	20 ± 3.3
**Exposed**	E31	2.2 ± 0.1			31 ± 3.0	
**Exposed**	G32	25 ± 0.8	7.4 ± 3.1			
**Exposed**	L33	16 ± 0.6				
**Protected**	C34					
**Protected**	C35					
**Protected**	D36					
**Exposed**	Q37	16 ± 2.1	5.4 ± 1.3			
**Protected**	C38					

**Protected**	R39					
**Protected**	F40					
**Protected**	M41					
**Exposed**	K42	3.6 ± 0.5	8.9 ± 1.2			
**Becomes exposed**	E43			6.0 ± 1.1	7.5 ± 1.6	9.0 ± 2.0
**Protected**	G44					
**Protected**	T45					
**Becomes exposed**	V46			6.3 ± 2.6		16.5 ± 5.0
**Protected**	C47					
**Protected**	R48					
**Exposed**	R49	7.9 ± 0.7	12 ± 2.0			
**Becomes exposed**	A50			4.0 ± 0.7	11 ± 2.0	8.4 ± 1.4
**Exposed**	R51	12 ± 1.4	14 ± 3.0			
**Exposed**	G52	13 ± 1				
**Exposed**	D53	32 ± 4	6.6 ± 1.7			
**Becomes exposed**	D54			8.0 ± 1.7	14 ± 1.1	15 ± 1.2
**Protected**	M55					
**Exposed**	D56	4.4 ± 0.6		8.0 ± 0.9		6.0 ± 1.4
**Protected**	D57					
**Protected**	Y58					
**Protected**	C59					
**Becomes exposed**	N60				13 ± 4.0	

**Protected**	G61					
**Protected**	I62					
**Protected**	S63					
**Exposed**	A64	3.4 ± 0.3	11 ± 2.0			
**Becomes exposed**	G65			9.0 ± 0.6	7.0 ± 1.3	
**Exposed**	C66	39 ± 9				
**Proline**	P67					
**Protected**	R68					
**Exposed**	N69	4.9 ± 1.7	4.1 ± 1.0			
**Proline**	P70					
**Protected**	F71					
**Protected**	H72					
**Protected**	A73					

Table S3. Correlation between the residues mapped as part of the binding cleft to the integrin αVβ3 (29) and the water exchanger behavior at pHs 6.0 (Table S1) and 7.5 (Table S2). The residues in red are the ones that were mapped as participating at the binding site. The residues in black are the fast exchanger residues vicinal to the residues participating in the binding. Note that most of the residues in the binding cleft are fast exchangers, especially at pH 7.5. Jarastatin binds at low affinity at pH 6.0 and at high affinity at pH 7.4 (K_D = 8 ± 1 nM).

Mapped Binding Cleft	pH 6.0	pH 7.5
A2	FAST EXCHANGER	FAST EXCHANGER
G3	FAST EXCHANGER	FAST EXCHANGER
E4		FAST EXCHANGER
E5	BECOMES EXPOSED	FAST EXCHANGER
C6	BECOMES EXPOSED	FAST EXCHANGER
D7	FAST EXCHANGER	FAST EXCHANGER
G9	FAST EXCHANGER	FAST EXCHANGER
T10		FAST EXCHANGER
G12	FAST EXCHANGER	FAST EXCHANGER
N13	BECOMES EXPOSED	FAST EXCHANGER
C16		FAST EXCHANGER
A18	BECOMES EXPOSED	FAST EXCHANGER
A19		FAST EXCHANGER
C21		FAST EXCHANGER
G26	FAST EXCHANGER	FAST EXCHANGER
E31		FAST EXCHANGER
G32		FAST EXCHANGER
L33	FAST EXCHANGER	FAST EXCHANGER
Q37	BECOMES EXPOSED	FAST EXCHANGER
K42		FAST EXCHANGER
R48	PROTECTED	PROTECTED
R49		FAST EXCHANGER
A50	BECOMES EXPOSED	BECOMES EXPOSED
R51		FAST EXCHANGER

**G52**	FAST EXCHANGER	FAST EXCHANGER
**D53**	FAST EXCHANGER	FAST EXCHANGER
**D54**	BECOMES EXPOSED	BECOME EXPOSED
**M55**	PROTECTED	PROTECTED
**D56**	BECOMES EXPOSED	FAST EXCHANGER
**Y58**	PROTECTED	PROTECTED
**A64**		FAST EXCHANGER
**C66**	FAST EXCHANGER	FAST EXCHANGER
**R68**	PROTECTED	PROTECTED
**N69**	BECOMES EXPOSED	FAST EXCHANGER
**F71**	PROTECTED	PROTECTED
**H72**	PROTECTED	PROTECTED
**A73**	PROTECTED	PROTECTED
